# Supplementary material for: Presenting a comprehensive multi-scale evaluation framework for participatory modelling programs: A scoping review
Source: PLoS One. 2022 Apr 22;17(4):e0266125. doi: 10.1371/journal.pone.0266125 (PMC9032404; doi:10.1371/journal.pone.0266125)

PLOS ONE

Presenting a comprehensive multi-scale evaluation framework for participatory modelling programs: A scoping review

--Manuscript Draft--

**Manuscript Number:**	PONE-D-21-25695
**Article Type:**	Research Article
**Full Title:**	Presenting a comprehensive multi-scale evaluation framework for participatory modelling programs: A scoping review
**Short Title:**	Comprehensive multi-scale evaluation framework for participatory modelling
**Corresponding Author:**	Grace Yeeun Lee<br>The University of Sydney<br>Camperdown, AUSTRALIA
**Keywords:**	Participatory modelling; evaluation; systems modelling and simulation; dynamic simulation model; stakeholder-based modelling; review; policy
**Abstract:**	Introduction<br><br>We live in a complex world with challenges that affect all aspects of our lives. Systems modelling and simulation can improve understanding and management of complex systems challenges. Advances in technology have facilitated accessibility of modelling by diverse stakeholders, allowing them to engage with and contribute to the development of systems models (participatory modelling). However, despite its increasing applications across a range of disciplines, there is a growing need to improve evaluation efforts to effectively report on the quality, importance, and value of participatory modelling.<br><br>Materials and Methods<br><br>A scoping review approach was utilized, which involved a systematic literature search via Scopus in consultation with experts to identify and appraise records that described an evaluation framework, criteria and/or process in the context of participatory modelling. This scoping review is registered with the Open Science Framework.<br><br>Results The review identified 11 studies, which varied in evaluation purposes, terminologies, levels of evaluation, and time points. The synthesis of studies highlighted areas of overlap and opportunities for further development, which prompted the development of a comprehensive multi-scale evaluation framework to assess participatory modelling programs across disciplines and systems modelling methods. The framework consists of four categories (Feasibility, Value, Change/Action, Sustainability) with 25 evaluation criteria, broken down across project-, individual-, group- and system-level impacts.<br><br>Discussion & Conclusion<br><br>The presented novel framework brings together a significant knowledge base into a flexible, cross-sectoral evaluation effort that considers the whole participatory modelling process. Developed through the rigorous synthesis of multidisciplinary expertise from existing studies, the application of the framework can provide the opportunity to understand practical future implications such as which aspects are particularly important for policy decisions, community learning, and the ongoing improvement of modelling methods.
**Order of Authors:**	Grace Yeeun Lee
	Ian Bernard Hickie
	Jo-An Occhipinti
	Yun Ju Christine Song
	Adam Skinner

Powered by Editorial Manager® and ProduXion Manager® from Aries Systems Corporation

	Salvador Camacho
	Kenny Lawson
	Adriane Martin Hilber
	Louise Freebairn
**Additional Information:**	
**Question**	**Response**
**Financial Disclosure**<br><br>Enter a financial disclosure statement that describes the sources of funding for the work included in this submission. Review the submission guidelines for detailed requirements. View published research articles from *PLOS ONE* for specific examples.<br><br>This statement is required for submission and **will appear in the published article** if the submission is accepted. Please make sure it is accurate.<br><br>**Unfunded studies**<br>Enter: *The author(s) received no specific funding for this work.*<br><br>**Funded studies**<br>Enter a statement with the following details:<br>• Initials of the authors who received each award<br>• Grant numbers awarded to each author<br>• The full name of each funder<br>• URL of each funder website<br>• Did the sponsors or funders play any role in the study design, data collection and analysis, decision to publish, or preparation of the manuscript?<br>• **NO** - Include this sentence at the end of your statement: *The funders had no role in study design, data collection and analysis, decision to publish, or preparation of the manuscript.*<br>• **YES** - Specify the role(s) played.<br><br>* typeset	This research is being conducted under the Brain and Mind Centre's Right care, first time, where you live Program, enabled by a AUD12.8 million partnership with BHP Foundation. The Program will develop infrastructure to support decisions relating to advanced mental health, and guide investments and actions to foster the mental health and wellbeing of young people in their communities.
**Competing Interests**<br><br>Use the instructions below to enter a competing interest statement for this submission. On behalf of all authors, disclose any competing interests that	Dr Louise Freebairn is currently employed part-time by the Brain and Mind Centre, University of Sydney; ACT Health as Director of Knowledge Translation and Health Outcomes, Epidemiology Section, ACT Health & CSART as Director of Policy Applications & Translational Science.<br><br>A/Professor Jo-An Occhipinti is both Head of Systems Modelling, Simulation & Data

could be perceived to bias this work—acknowledging all financial support and any other relevant financial or non-financial competing interests.<br><br>This statement is required for submission and **will appear in the published article** if the submission is accepted. Please make sure it is accurate and that any funding sources listed in your Funding Information later in the submission form are also declared in your Financial Disclosure statement.<br><br>View published research articles from *PLOS ONE* for specific examples.<br><br>**NO authors have competing interests**<br><br>Enter: *The authors have declared that no competing interests exist.*<br><br>**Authors with competing interests**<br><br>Enter competing interest details beginning with this statement:<br><br>*I have read the journal's policy and the authors of this manuscript have the following competing interests: [insert competing interests here]*<br><br>* typeset	Science at the University of Sydney's Brain and Mind Centre and Managing Director of Computer Simulation & Advanced Research Technologies (CSART).<br><br>Professor Ian Hickie is the Co-Director, Health and Policy at the Brain and Mind Centre (BMC) University of Sydney. The BMC operates an early-intervention youth services at Camperdown under contract to headspace. He is the Chief Scientific Advisor to, and a 5% equity shareholder in, InnoWell Pty Ltd. InnoWell was formed by the University of Sydney (45% equity) and PwC (Australia; 45% equity) to deliver the $30 M Australian Government-funded Project Synergy (2017-20; a three-year program for the transformation of mental health services) and to lead transformation of mental health services internationally through the use of innovative technologies.
**Ethics Statement**<br><br>Enter an ethics statement for this submission. This statement is required if the study involved:<br><br>• Human participants<br>• Human specimens or tissue<br>• Vertebrate animals or cephalopods<br>• Vertebrate embryos or tissues<br>• Field research<br><br>Write "N/A" if the submission does not require an ethics statement.	N/A

Yes - all data are fully available without restri

General guidance is provided below. Consult the submission guidelines for detailed instructions. **Make sure that all information entered here is included in the Methods section of the manuscript.**<br><br>**Format for specific study types**<br><br>**Human Subject Research (involving human participants and/or tissue)**<br>• Give the name of the institutional review board or ethics committee that approved the study<br>• Include the approval number and/or a statement indicating approval of this research<br>• Indicate the form of consent obtained (written/oral) or the reason that consent was not obtained (e.g. the data were analyzed anonymously)<br><br>**Animal Research (involving vertebrate animals, embryos or tissues)**<br>• Provide the name of the Institutional Animal Care and Use Committee (IACUC) or other relevant ethics board that reviewed the study protocol, and indicate whether they approved this research or granted a formal waiver of ethical approval<br>• Include an approval number if one was obtained<br>• If the study involved *non-human primates*, add *additional details* about animal welfare and steps taken to ameliorate suffering<br>• If anesthesia, euthanasia, or any kind of animal sacrifice is part of the study, include briefly which substances and/or methods were applied<br><br>**Field Research**<br><br>Include the following details if this study involves the collection of plant, animal, or other materials from a natural setting:<br>• Field permit number<br>• Name of the institution or relevant body that granted permission	
**Data Availability**<br><br>Authors are required to make all data	Yes - all data are fully available without restriction

underlying the findings described fully available, without restriction, and from the time of publication. PLOS allows rare exceptions to address legal and ethical concerns. See the PLOS Data Policy and FAQ for detailed information.<br><br>A Data Availability Statement describing where the data can be found is required at submission. Your answers to this question constitute the Data Availability Statement and **will be published in the article**, if accepted.<br><br>**Important:** Stating 'data available on request from the author' is not sufficient. If your data are only available upon request, select 'No' for the first question and explain your exceptional situation in the text box.<br><br>Do the authors confirm that all data underlying the findings described in their manuscript are fully available without restriction?	
**Describe where the data may be found in full sentences. If you are copying our sample text, replace any instances of XXX with the appropriate details.**<br><br>• If the data are **held or will be held in a public repository**, include URLs, accession numbers or DOIs. If this information will only be available after acceptance, indicate this by ticking the box below. For example: *All XXX files are available from the XXX database (accession number(s) XXX, XXX.*).<br>• If the data are all contained **within the manuscript and/or Supporting Information files**, enter the following: *All relevant data are within the manuscript and its Supporting Information files.*<br>• If neither of these applies but you are able to provide **details of access elsewhere**, with or without limitations, please do so. For example:<br><br>*Data cannot be shared publicly because of [XXX]. Data are available from the*	The authors have disclosed and detailed the search strategy of the scoping review, which can be replicated.

XXX Institutional Data Access / Ethics Committee (contact via XXX) for researchers who meet the criteria for access to confidential data.<br><br>*The data underlying the results presented in the study are available from (include the name of the third party and contact information or URL).*<br>• This text is appropriate if the data are owned by a third party and authors do not have permission to share the data.<br><br>* typeset	
Additional data availability information:	

Manuscript of scoping review
Click here to access/download;Manuscript;Lee GY_PM evaluation framework scoping review_FINAL.docx

Research article

Presenting a comprehensive multi-scale evaluation framework for participatory modelling programs: A scoping review

Grace Yeeun Lee,[1*] Ian Bernard Hickie,[1±] Jo-An Occhipinti,[1,2±] Yun Ju Christine Song,[1±] Adam Skinner,[1] Salvador Camacho,[3] Kenny Lawson,[1] Adriane Martin Hilber,[3] Louise Freebairn[1,2,4]

[1] Brain and Mind Centre, The University of Sydney. Sydney, NSW Australia.
[2] Computer Simulation & Advanced Research Technologies (CSART). Sydney, NSW Australia.
[3] Swiss Centre for International Health, Swiss Tropical and Public Health Institute. Basel, Switzerland.
[4] Research School of Population Health, The Australian National University. Canberra, ACT Australia.

*Corresponding author
[±]Equal contributions, listed in alphabetical order

Address: Brain and Mind Centre, 88 Mallett Street, Camperdown, NSW 2050, Australia
Email: grace.lee@sydney.edu.au
Phone: [+61 481 964 231]

Abstract

long abstract

Introduction

We live in a complex world with challenges that affect all aspects of our lives. Systems modelling and simulation can improve understanding and management of complex systems challenges. Advances in technology have facilitated accessibility of modelling by diverse stakeholders, allowing them to engage with and contribute to the development of systems models (participatory modelling). However, despite its increasing applications across a range of disciplines, there is a growing need to improve evaluation efforts to effectively report on the quality, importance, and value of participatory modelling.

add objective(-s)

Materials and Methods

A scoping review approach was utilized, which involved a systematic literature search via Scopus in consultation with experts to identify and appraise records that described an evaluation framework, criteria and/or process in the context of participatory modelling. This scoping review is registered with the Open Science Framework.

Results

The review identified 11 studies, which varied in evaluation purposes, terminologies, levels of evaluation, and time points. The synthesis of studies highlighted areas of overlap and opportunities for further development, which prompted the development of a comprehensive multi-scale evaluation framework to assess participatory modelling programs across disciplines and systems modelling methods. The framework consists of four categories (*Feasibility, Value, Change/Action, Sustainability*) with 25 evaluation criteria, broken down across project-, individual-, group- and system-level impacts.

synthesis?

Discussion & Conclusion

The presented novel framework brings together a significant knowledge base into a flexible, cross-sectoral evaluation effort that considers the whole participatory modelling process.

Developed through the rigorous synthesis of multidisciplinary expertise from existing studies, the application of the framework can provide the opportunity to understand practical future implications such as which aspects are particularly important for policy decisions, community learning, and the ongoing improvement of modelling methods.

Develops a framework based on 11 other frameworks.

”Synthesis of expertise” = the frameworks. There are so much knowledge represented by/in the frameworks. This can not be captured by a word-analysis solely.

Keywords

Participatory modelling, evaluation, systems modelling and simulation, dynamic simulation model, stakeholder-based modelling, review, policy

Introduction

Traditional *versus* Participatory modelling

Systems modelling and simulation, also known as dynamic simulation modelling, is a term given to complex systems science analytic methods – such as system dynamics, Bayesian networks, and agent-based models – that is utilized in many countries and across diverse sectors to support evidence-informed decision making and to drive policy reform.[1, 2] By taking a complex systems view, significant challenges in society including population health crises, climate change, poverty, and civil strife can be better understood and managed through computer simulation models that capture the causal structure underlying the dynamics of these systems.[1, 3-8] Various systems modelling and simulation techniques have traditionally been applied across a range of disciplines including engineering, business, and environmental sciences for decades,[9] but is now increasingly utilized in other fields including in public health.[10-12] This is largely attributed to the utility of systems modelling and simulation providing decision makers with both immediate and long-term support in understanding the prospective impacts of alternative strategic actions, where traditional statistical methods may be limited.[13-16]

Systems modelling and simulation can provide insights at different levels of scale, including macro, meso, and micro; providing national, state, and local governments with tools that support strategic planning and decision making.[4, 17-20] As national models can often hide significant regional variations, a key strength of the models developed for local decision making is that they can be contextualized to the complex, local system of interest.[17, 21] The need to ground models in local context is giving rise to an increasing commitment to the inclusion of stakeholders in the model building process.[21]

Participatory modelling (PM), or stakeholder-based systems modelling, brings together scientific and local expert knowledge. Advances in technology and software have facilitated

By your explanation of PM it seams that the model development or use is the focus. Not a planning/decision-making process that includes (participatory) modeling. If you want a definition that includes both perspectives - see reference [29]

Focus on participatory model use and development, as such, or, on participatory planning and decision-making (including (participatory) modelling)? Be clear about which because it makes a great difference.

accessibility of modelling by a broader group of participants, allowing more diverse stakeholders working across a complex system to engage with and contribute to the development of these models.[11, 22] For example, graphical model interfaces allow stakeholders to better visualize and understand the logic and assumptions of a model than earlier software that required articulation of a model using mathematical equations or computer coding. Such accessibility has also facilitated the participation of those most impacted by policy changes (such as consumer representatives) – helping to work towards all stakeholders sharing a common understanding of a complex problem or issue, inform and enhance collective action, assist collective decision making processes, enhance both individual and social learning, as well as precipitate changes in stakeholder behaviors.[9, 21, 23-29]

Evaluating participatory modelling studies

The inclusion of stakeholders to inform the model building process has led to changes in evaluation practices to reflect this participatory approach.[30-32] By drawing on diverse stakeholder input, experiences of the quality, importance, and value of PM can be better understood. Common evaluation approaches include *formative, summative, process, impact* and *outcome* evaluation.[33] These evaluation approaches can be applied individually or in combination depending on the program or program activity being evaluated. *Formative evaluations* aim to strengthen the implementation of a program or activity before it is fully implemented, and is typically conducted when the program or activity is being developed or modified;[33-35] *summative evaluations* aim to demonstrate whether the program or activity achieved its intended outcomes to understand its ultimate value, and is conducted towards the end of a program;[34, 36] *process evaluations,* similar to formative evaluations, are embedded as part of implementation, and study the processes or factors related to successful implementation of the program or activity for its intended purpose;[33, 35, 37] *impact evaluations* assess whether the immediate outcomes are attributable to the program or

This section is mainly about evaluation - not evaluation of participatory modelling studies.

Also - much general info about evaluation that is not clearly directed towards the study's standpoint on evaluation.

activity,[36, 38] and; *outcome evaluations* measure the longer-term outputs of the program or activity, and are commonly measured retrospectively.[33, 37]

At the most basic level, evaluations provide systematic comparisons of program objectives and outcomes to understand how well something is working for the purpose of policy, planning, or implementation.[38, 39] According to the Cambridge Dictionary, evaluation is defined as the *"process of judging the quality, importance, amount, or value of something."*[40] Applying this definition to the context of this paper, there is opportunity to better understand the quality, importance, and value of PMs.[37] This shifts the focus from solely the technical model to a more holistic consideration of the whole PM process, providing opportunity for further knowledge on which aspects of PM are particularly important for policy decisions and community learning, as well as the ongoing improvement of PM methods.[41, 42]

Evaliuation can also focus on understanding why (explaining) something works as intended or not.

Evaluators are relied upon to address questions on the effectiveness of investments in local, state, and national programs.[43] There may be various motivations for conducting an evaluation of PM programs including the desire to improve and share knowledge on good practice for PM, quantitatively and qualitatively report on project impacts, as well as to assess the value of PM for future work.[23, 37] Evaluations also keep the modellers, funders, and other stakeholders of interest held accountable for demonstrating outcomes, as well as to provide merit to the work being evaluated.[23] Thus, PM program evaluations can also support policy makers to make evidence-informed decisions in determining how much weight to give the program or model outputs.[23]

Challenges and opportunities in participatory modelling evaluation

There is growing interest in the field of PM evaluation; however, in most cases, evaluation is "lacking or is not based on transparent and systematic methodological approaches."[44] It is

also acknowledged that evaluations that comprehensively capture the complex nature of PM can be difficult,[45] as embedding participatory approaches in systems modelling and simulation creates several challenges.[37] For instance, the focus of PM outcomes is often still on the final technical model itself rather than the participatory process used to develop the models.[23] Additionally, previous studies that have attempted to evaluate the benefits of PM have reported difficulty in the design of the evaluation process, as complex systems rarely have comparative controls that allow for feasible experimental design (i.e., 'with modelling' intervention *vs* 'without modelling' control), making the measure of PM effectiveness challenging.[37] Thus, it is important to understand the distinction between the evaluation in detecting the effectiveness of the model development process, compared to the actual success or failure of the engagement with the model itself.

PM evaluations are also typically constrained by contextual factors including limited time and program budget. PM programs are often funded to the point of delivering the final model, rather than evaluating the process and benefits of PM including the extent to which the final model informed decision making or built consensus. A lack of investment in the evaluation of PM leads to decreased motivation to conduct thorough evaluations and may also risk evaluation efforts to be overly simplified when measuring the impact of PM, [23] missing the opportunity to assess the performance of PM in different contexts to inform the adaption and improvement of processes.[46]

Objectives

Therefore, this scoping review aims to:

1. Identify published PM evaluation frameworks, criteria and/or processes irrespective of the modelling method, or the discipline in which they are designed and/or implemented.

focus on different things in an evaluation. Claims of comprehesiveness may generate false believes of that the evaluation may provide the "only true" understanding of PM while every effort has its underlying beliefs/ theoretical perspective etc. Important to acknowledge that, and try to be clear with what perspective(s) the evaluative framework aid to make visible/ measures /assesses

Good (clear and relevant) description of objectives.

2. Assess the identified evaluation frameworks, criteria and/or processes to understand their applicability to different PM program objectives and contexts.
3. Synthesize the findings to develop an evaluation framework that can be adapted and executed in diverse PM programs, regardless of the discipline or modelling method.

A scoping review has been deemed the most appropriate approach, compared to a systematic review, as the purpose of this paper is to focus on the broad collection and discussion of available literature, and to present a comprehensive multi-scale evaluation framework for PM programs.[47, 48] The development and application of the presented evaluation framework is supported by a participatory systems modelling program for youth mental health (described elsewhere). To our knowledge, this is the first multidisciplinary scoping review of evaluation frameworks for PM programs.

Please refer to this study, and tell a little bit more about it (or take it away).

Agree, to my knowledge too

Materials and Methods

This scoping review was conducted according to the suggested methodology outlined in the *Joanna Briggs Institute (JBI) Reviewers' Manual for Evidence Synthesis,*[49] in combination with additional recommendations for conducting scoping reviews.[50] The Preferred Reporting Items for Systematic Reviews and Meta-Analyses (PRISMA) statement was also applied.[51] This review paper has also been registered with the Open Science Framework.[52]

Search strategy

A focused search was conducted via Scopus in May 2021 in consultation with an academic librarian at The University of Sydney, utilizing a combination of Boolean operators, wildcards, truncation and Medical Subject Headings to develop the final search strategy (Table 1). Scopus is a meta-database, and includes records from various databases across disciplines including environmental sciences, engineering, mathematics, social sciences and medicine.[41] Additional

searches were conducted through hand searching, co-author recommendations and citation chaining.

Table 1. Search strategy.

**Topic**	**Searches**	**Results**
**Scopus**		
(Participatory) Systems modelling and simulation	(TITLE-ABS-KEY (“simulation model*”)) OR (TITLE-ABS-KEY (“system* model*”)) OR (TITLE-ABS-KEY (“participator* model*”)) OR (TITLE-ABS-KEY (“system* dynamic*”)) OR (TITLE-ABS-KEY (“agent-based model*”)) OR (TITLE-ABS-KEY (“discrete event simulation”)) OR (TITLE-ABS-KEY (“Bayesian network*”)) OR (TITLE-ABS-KEY (“hybrid simulation”)) OR (TITLE-ABS-KEY (“system* science”)) OR (TITLE-ABS-KEY (“stakeholder-based model*”))	256,087
Participatory design	(TITLE-ABS-KEY (“participator*)) OR (TITLE-ABS-KEY (co-design*))	89,247
Evaluation	(TITLE-ABS-KEY (evaluat*)) OR (TITLE-ABS-KEY (“review*))	13,380,297
Combined queries		465
**Additional search**		
Hand searching, co-author recommendations, citation chaining		10
**Total yielded literature**		**475**

Inclusion and exclusion criteria

very good description of the search and selection process

The criteria for inclusion were defined a priori by the authors (GYL, LF) in a Population, Concept, Context format,[49] and applied to all yielded records. As detailed in Table 2, this scoping review included sources that described an evaluation framework, criteria, and/or process for PMs. Though there are varying definitions that exist, for the purpose of this review, we have defined an *evaluation framework* as a tool that presents an overview of the evaluation theory, topics or themes, questions, and/or data sources; *evaluation criteria* as a performance metric or indicator that further breaks down the evaluation framework, and; *evaluation process* as a defined evaluation procedure guided by theory of how the authors recommend PM.[53]

Records that presented a standalone theoretical framework (or applied via a case study example) were also included. In contrast, records that only described the methodological tools (e.g., interviews, etc) used to evaluate the implementation of PM programs without describing an evaluation framework, criteria and/or process were excluded. Records that only

described the evaluation of a technical model (e.g., not PM) were excluded, as were records that described PM implementation programs without any consideration of evaluations. Date limits were not set, but studies not published in English were excluded from the review.

Table 2. Inclusion and exclusion criteria in a Population, Concept, Context format, as recommended by JBI.

	**Inclusion criteria**	**Exclusion criteria**
**Population**	Not defined (due to the limited number of PM evaluation frameworks, criteria and/or processes, broadened the 'population' category to not be defined to specific fields of disciplines/population groups).	
**Concept (e.g., PM program methods)**	Records describing an evaluation framework, criteria and/or process to support the evaluation of a PM program (standalone theoretical framework, or applied in a case study).	Records solely describing the methods adopted to evaluate the implementation of PM programs, without describing an evaluation framework, criteria and/or process.<br><br>Records solely describing the evaluation of the technical model (e.g., not PM).<br><br>Records describing PM implementation programs not evaluated.
**Context (e.g., country, setting)**	Not defined (due to the limited number of PM evaluation frameworks, criteria and/or processes, broadened the 'context' category to not limit any cultural, geographic, or specific setting factors).	Outcomes not published in English.

Data extraction and synthesis

Using a pro forma approach, the first author (GYL) independently reviewed the titles and abstracts of all yielded records. Uncertainty whether records met the inclusion criteria were resolved through two-weekly discussions with the senior author (LF). To verify the data extraction, a random sample of 10 records were independently checked by LF. Following this verification process, full text review and data extraction was conducted independently by GYL.

To address the first and second objective, a data extraction template was developed by the authors (GYL, LF) and used to collate information on yielded records that underwent full text

review. The four-dimensional framework (4P) developed by Gray *et al.* was used to set the basis of the data extraction template. The 4P framework is novel as it was developed specifically to standardize the communication of reporting PM programs.[42] This framework has since been adapted to include two additional dimensions by Freebairn – imPact and Prioritizing.[13] The resulting six dimensions (6P) of the adapted framework include: *Purpose* (why participatory approaches should be adopted in PM); *Process* (how stakeholders were engaged to collaboratively build the systems model); *Partnerships* (who the stakeholders involved were and why); *Products* (what the outcomes were as a result of adopting the PM approach); *imPact* (what impacts resulted from the PM process, including knowledge exchange and capacity building, future forecasting, and policy decision making), and; *Prioritizing* (what future priorities were identified as a result the PM process).[13] The 6P definitions were adapted to fit the evaluation objectives of this scoping review, as detailed in Table 3. To ensure an all-inclusive synthesis of records, the JBI template for data extraction[49] as well as additional elements included by the authors were also incorporated into the final data extraction template (Table 3). Once the author (GYL) completed full text review, the senior author (LF) reviewed and verified the final list of records to include for synthesis.

Table 3. Data extraction template.

Source	Data extraction criteria
JBI	Reference<br>Author, year of publication, title, journal, volume, issue, pages
JBI	Population
JBI	Concept (e.g., PM program methods)
JBI	Context (e.g., country, setting)
6P	Purpose (e.g., why PM approaches should be evaluated)
6P	Process (e.g., method utilized to execute evaluation framework/criteria/process)
6P	Partnerships (e.g., stakeholders involved in the development of the evaluation framework/criteria/process)
6P	Products (e.g., level of evaluation – theoretical, conceptual, implementation)
6P	imPact (e.g., outcomes/strengths of the evaluation framework/criteria/process)
6P	Prioritizing (e.g., barriers, future opportunities of the evaluation framework/criteria/process)
GYL, LF	Other important themes?

GYL, LF	Evaluation framework criteria
GYL, LF	Include in scoping review?
GYL, LF	Other comments

**JBI* refers to the Joanna Briggs Institute's Reviewers' Manual for Evidence Synthesis; *6P* refers to Freebairn's adapted framework to standardize reporting of PM programs; *GYL, LF* refers to additional elements included by the first and senior authors to enable an all-inclusive data extraction process.

To address the third objective, a narrative synthesis of the findings was conducted and utilized to develop an evaluation framework that can be applied across diverse disciplines and modelling methods.

What's a narrative synthesis? Please describe

Results

Study selection

The initial Scopus search yielded 465 results; an additional 10 records were identified through hand searching, co-author recommendations, and citation chaining. Most articles were excluded from review based on their titles and abstracts (n=451), as the majority only vaguely described the evaluation methods or outcomes of the implementation of a PM program without any reference to an evaluation framework, criteria and/or process. After screening 24 full-text records, 11 studies were included for synthesis. Though it was not intentional, all included records were from academic journals, as opposed to grey literature and conference papers. The PRISMA flow diagram is presented in Figure 1.

[Suggested location of figure]

Figure 1. PRISMA flow diagram.

Characteristics of studies

Table 4 summarizes the characteristics of the studies. All but one (10/11, 90.9%) were published in an environmental sciences journal. The remaining one study was published in the International Journal of Environmental Research and Public Health. Approximately half (7/11, 63.6%) of the included studies were published in the past decade (2011-2021). In

comparison, three quarters (346/475, 72.8%) of the 475 total yielded records reviewed by their abstracts and titles were published in the last decade.

Table 4. Characteristics of included studies.

**First author**	**Year of publication**	**Name of article**	**Academic journal published**
**Lynam**[54]	2007	A Review of Tools for Incorporating Community Knowledge, Preferences, and Values into Decision Making in Natural Resources Management	Ecology and Society
Jones[24]	2009	Evaluating Participatory Modelling: Developing a Framework for Cross-Case Analysis	Environmental Management
Zorrilla[55]	2010	Evaluation of Bayesian Networks in Participatory Water Resources Management, Upper Guadiana Basin, Spain	Ecology and Society
Matthews[56]	2011	Raising the bar? The challenges of evaluating the outcomes of environmental modelling and software	Environmental Modelling & Software
Smajgl[57]	2015	Evaluating participatory research: Framework, methods and implementation results	Journal of Environmental Management
Maskrey[58]	2016	Participatory modelling for stakeholder involvement in the development of flood risk management intervention options	Environmental Modelling & Software
Falconi[37]	2017	An interdisciplinary framework for participatory modelling design and evaluation – What makes models effective participatory decision tools?	Water Resources Research
Hedelin[59]	2017	Participatory modelling for sustainable development: Key issues derived from five cases of natural resource and disaster risk management	Environmental Science and Policy
**Hamilton**[23]	2019	A framework for characterizing and evaluating the effectiveness of environmental modelling	Environmental Modelling & Software
Waterlander[60]	2020	A System Dynamics and Participatory Action Research Approach to Promote Healthy Living and a Healthy Weight among 10–14-Year-Old Adolescents in Amsterdam: The LIKE Programme	International Journal of Environmental Research and Public Health
Zare[61]	2021	A formative and self-reflective approach to monitoring and evaluation of interdisciplinary team research: An integrated water resource modelling application in Australia	Journal of Hydrology

Bold and italics = clear evaluation framework and/or criteria defined, with case study described; **Bold = clear evaluation framework and/or criteria defined, with no case study**; *italics = evaluation process defined, with case study described*

Characteristics of evaluation frameworks, criteria and/or processes

The papers included for synthesis either described a theoretical evaluation framework and/or criteria with no application to a case study;[23, 54] described a theoretical evaluation framework and/or criteria applied to a case study;[24, 37, 55, 58, 59] or; described an evaluation process applied to a case study.[56, 57, 60, 61] The majority of the evaluation frameworks, criteria and/or processes were developed by building upon already existing work.[24, 37, 55-61] Only two of the evaluation frameworks described an empirical process of how their frameworks were developed, supplemented with literature reviews.[23, 54] The evaluation frameworks, criteria, and/or processes focused on different design features and levels of examination. For example, Jones *et al.*, Hamilton *et al.*, Zare *et al.*, and Smajgl & Ward noted the importance of evaluation both *ex ante* and *ex post* to ensure all involved – including modellers and stakeholders – have a better understanding of what the PM process is aiming to achieve, and to keep everyone accountable to the defined objectives.[23, 24, 57, 61] Jones *et al.* and Hamilton *et al.* also differentiated between the different levels of stakeholder participation;[23, 24] and although this was not embedded into the evaluation criteria presented by Zorrilla *et al.*, there was consideration that future work should break down evaluation amongst stakeholder groups from policy makers to farmers.[55] There were two papers that did not explicitly consider the different levels of impact of PM (e.g., project-level impact *vs* system-level impact).[54, 56] Maskrey *et al.*, Falconi *et al.*, and Hamilton *et al.* recognized that evaluations should also consider both the immediate and long-term outcomes.[23, 37, 58] Hedelin *et al.* and Waterlander *et al.* focused on the organizational level – for example, focusing on the various elements of the system to understand organizational learning, change, and action.[59, 60] This information is summarized in Table 5.

For the papers 24, 37, 55-61 - Did you look up the references to the ”existing work”? The theoretical or empirical grounds for these frameworks should be described there…

Table 5. Characteristics of evaluation frameworks, criteria and/or processes reported through individually included studies.

**First author (Year)**	**Brief description of evaluation framework, criteria and/or process**	**Method of evaluation framework, criteria and/or process development**	**Application of evaluation framework, criteria and/or process**	**Level of evaluation** Possible to find a better word than "Level" here? Not very descriptive… Perhaps Target point? You seem to capture a lot of different things here…	**Strengths and limitations**
**Lynam (2007)**	Framework and criteria presented to evaluate various PM tools (e.g. Bayesian belief network, systems models, etc) in the natural resource management context. Three categories of evaluation criteria: 1) *Capabilities,* evaluating potential applications of the participatory tool, 2) *Use*, evaluating conditions or context of use, and 3) *Products*, evaluating the nature of the results/outputs of the tools. Full criteria presented in *Tables 2, 3* and *4*.	Not described at length, but a discussion-based process amongst authors, with cross-examination with the literature to validate discussions briefly described.	Authors have stated that the decision to not demonstrate the application of the evaluation framework with field examples was intentional, with the justification that the amount of text needed to provide adequate context for each PM tool outweighed the benefits.	N/A – Not explicitly mentioned Perhaps remove this column? Or, make clear its target. Define it before using it in this table.	*Strengths.* One of the first academic papers that considered systematic evaluation approaches in PM, and considers both the process (e.g., co-learning), as well as the technical model (e.g., reasonable levels of accuracy). Enables others to adopt the most appropriate PM tool by considering strengths and limitations presented. *Limitations.* Lack of empirical evidence regarding the development of the presented evaluation framework and criteria.

Jones (2009)	Evaluation framework (named *Protocol of Canberra*) developed in the context of natural resource management to assess extent to which different PM initiatives not only modify perceptions, but also facilitate change in interactions between participants to make collective decisions. Consists of two main components: 1) *Designers Questionnaire,* which captures the project team's experiences, and 2) *Participants Evaluation Guide,* which captures the participants' experiences of the project.	Developed through a collaboration between French and Australian researchers in 2005 (ADD-ComMod project) engaged in PM and evaluation research. Framework designed to identify *context* (e.g., setting)*, process* (e.g., method), and *underlying theoretical thread* (e.g., theory-based evaluation to understand link between theory and practice, building off work of Argyris,[62] Patton[63] and Curnan).[64]	Presented three case studies as part of the ADD-ComMod evaluation project, to demonstrate the use of the evaluation framework in different contexts: 1) *AtollGame,* ex post evaluation of groundwater management in the Republic of Kiribati, 2) *Catalyst Project,* ex post evaluation of strategic regional development planning conducted by CSIRO, and 3) *Lower Hawkesbury Estuary Management Plan,* ongoing process evaluation to manage the Lower Hawkesbury Estuary in Australia.	Level of evaluation differentiated by research participants *vs* project team. Can be applied as either an *ex post* (conducted after implementation) or ongoing evaluation (carried out during the project as it progresses).	*Strengths*. Evaluation framework is flexible to encompass various approaches to PM. *Limitations*: The authors of the evaluation framework admitted that the robustness of their evaluation framework has come at the cost of simplification – specifically, assumption of linear structure of framework.

Zorrilla (2010)	Process evaluation criteria for public participation (and its PM tools), with an emphasis on "what works best when" in the context of water resources management. Evaluation criteria broken down to the *participatory process* (e.g., improve system understanding, foster trust, etc), as well as *capabilities of Bayesian networks* (e.g., graphical interface, level of knowledge or uncertainty, etc).	Process evaluation followed methodology outlined by Abelson *et al.* (2003),[65] Rowe and Frewer (2004),[66] and Von Korff (2006).[67] Criteria used to evaluate the PM process itself (e.g., increase stakeholder understanding, foster trust, etc), as well as to evaluate the quality of Bayesian networks as a PM tool (e.g., graphical interface, etc). References to each evaluation criteria provided (*Tables 3* and 4); criteria to evaluate PM tool belongs to three categories described by Lynam *et al.,* 2007[54] – *capabilities, use* and *products*.	Evaluation criteria applied to evaluate the ability of Bayesian networks to effectively engage stakeholders and to support decision making in complex situations (challenges caused by uncontrolled groundwater extraction in Upper Guardiana Basin, Spain). Employed mixed methods: 1) *Stakeholder evaluation questionnaires,* 2) *Semi-structured telephone interviews,* 3) *Researchers' theoretical assessment* (Stewart *et al.,* 1984;[68] Einsidiel *et al.,* 2001;[69] Henriksen *et al*., 2007[70]).	Not clear. However, authors have recognized that future studies should break down level of evaluation amongst stakeholder groups (policy makers *vs* technicians, farmers, and environmentalists).	*Strengths.* Presentation of evaluation criteria which considers participatory process, as well as influence of the actual model itself. Evaluation framework is built on research evidence, with application and outcomes exemplified through a case study. *Limitations.* Authors have recognized the short length of the questionnaire may have been a limitation (i.e., more rigorous development of evaluation tools are required.

Matthews (2011)	Conceptual evaluation process described that situates outcome evaluation within the wider context of environmental modelling and software activity (EMS) to recognize the differentiation between *outcomes* (changes in values, attitudes and behavior) and *outputs* (knowledge mobilized in peer reviewed articles, software, or datasets). The conceptual evaluation process consists of three loosely coupled phases that link EMS research to outcomes – *research, development* and *operations* – in which evaluation plays an integral role across all phases.	Evaluation process built on understanding of the relationship between *context, process* and *outcomes* (Blackstock *et al.,* 2007,[71] and; Patton, 1998).[72] Conceptual evaluation process is a generalization of the "consultancy model" for successful Decision Support System proposed by McCown (2002),[73] where knowledge is passed between phases rather than software tools.	Applied evaluation process in a case study (Communicating Climate Change Consequences for Land Use, C4LU project). Outcome evaluation conducted utilizing different methodological approaches depending on the phase (e.g., *research* – peer review and validation; *development* – parallel process of software engineering and quality assurance, as well as workshops with national policy makers; *operations* – evaluation form and workshop recordings.	N/A – Not explicitly mentioned.	*Strengths.* Recognition of the importance of conceptual processes to be coupled with practical evaluation methods. *Limitations.* Evaluation process is arguably too simple with no details on evaluation framework or criteria. Authors have disclosed that while a simple evaluation of the C4LU workshop outcomes was conducted, more work is required on the design and interpretation of such evaluation processes.

Smajgl (2015)	Evaluation protocol to facilitate systems learning through a structured participatory process by decision makers concerned with the management of environmental resources.	Evaluation protocol built off of the Challenge and Reconstruct Learning (ChaRL) Framework (Smajgl and Ward, 2013),[74] which draws on theoretical concept of complex systems, decision making constructs, and influences of learning (e.g., values, beliefs and attitudes).	Evaluation of series of PM workshops where an agent-based simulation model was used to challenge existing beliefs concerned with the effectiveness of proposed policy actions and development ideas in the Greater Mekong Subregion. Employed a mixed methods (i.e., questionnaires and workshops); decision maker learning was evaluated via observed changes in individually held values, beliefs, and attitudes.	Not clear; participation of workshops was targeted for stakeholders that had some degree of influence to make decisions.	*Strengths.* Recognition of the importance of both *ex ante* and *ex post* evaluation approach. *Limitations.* Theoretical underpinnings of the evaluation protocol relies heavily on decision making evidence, rather than considering evidence on how to effectively evaluate PM as a whole (both process and technical model).
Maskrey (2016)	Evaluation framework designed to understand the benefits and limitations of the PM process itself, and assessment of outcomes. Evaluation framework executed via *process evaluation* (criteria broken down broken down across themes: *accessibility, deliberation, representation, responsiveness, satisfaction)* as well as *outcome evaluation* (broken down into *substantive outcomes* and *social outcomes*).	Developed from synthesis of frameworks by Beierle (1999),[75] Rowe and Frewer (2000),[76] and Webler and Tuler (2002).[77] Additional references provided for individual criterion in *Tables 7* and *9*.	Evaluation framework applied in a simple Bayesian network model to exemplify how PM can support local flood risk management contexts in Hebden Water catchment (UK).	Evaluation framework enables the consideration of the process and final outcome (e.g., short- *vs* long-term outcomes).	*Strengths.* Consideration of both short-term substantive and longer-term social benefits. Comprehensive evaluation criteria presented with clear disclosure of evidence base, as well as key findings from the Hebden Water catchment case study. *Limitations*. Though emphasis was made on short- *vs* long-term outcomes, not clear in case study whether sufficient time between evaluation points would have allowed for long-term outcomes reporting.

Falconi (2017)	Two-stage PM evaluation framework which aimed to improve model effectiveness as participatory tools through standardizing data, documentation, and reporting practices. *1) Stage one:* Five dimensions of public participation in environmental decision making: *participants, stages of participation, degree of involvement, level of influence, purpose (who, when, how, why),* and; *2) Stage two:* Attributes of successful PMs – categorized into three evaluation criteria: *credibility, salience, legitimacy.*	Stage one adapted from the National Resource Council (2008);[78] Stage two expanded characteristics described by Cash *et al.,* 2003[79] and Carr *et al.,* 2012.[80]	Application of evaluation criteria across five distinct case studies: 1) *Community-based forest management,* Zimbabwe, 2) *Shared vision modelling for ACT-ACF water basin*, USA, 3) *Water management alternatives*, USA, 4) *Water resource allocation*, Solomon Islands, and 5) *Regional land-use*, Senegal River Delta.	Consideration of intermediate outcomes (distinct from 'ultimate outcomes').	*Strengths.* Two-stage evaluation framework provides standardized mechanism that capture both the technical and social nature of PM. Flexibility to apply the framework in wide range of cases across disciplines. *Limitations*. By way of the authors demonstrating the applicability of their framework in five case studies, there was no comprehensive application of the framework (e.g., only narrative summaries described).
Hedelin (2017)	Evaluation framework presented with 16 criteria for procedural sustainable development, with key sustainability principles (*integration, participation*) applied across themes (*across disciplines; across values; contributing to the process; generating commitment, legitimacy or acceptance; across organizations)*	Development process not described; reader directed to Hedelin 2007,[81] Hedelin 2015,[82] and Hedelin 2016[83] for more information on theoretical basis of the evaluation framework.	Framework applied to five case studies on large PM projects that span from water management to flood risk management programs. Did not report on individual case studies but rather, themes of case studies applied to the evaluation framework.	Not clear; however, evaluation criteria derived from research on organization learning, multilevel governance, organization coordination, and collaborative planning.	*Strengths.* Evaluation framework developed for sustainable development research, but can be applied to other disciplines of research. *Limitations.* Despite the emphasis on decision support, evaluation criteria of the five case studies did not sufficiently report on decision support outcomes.

**Hamilton (2019)**	Comprehensive formative evaluation framework to consider multiple dimensions and perspectives in environmental PM studies. Evaluation criteria grouped into eight categories: *project efficiency, model accessibility, credibility, salience, legitimacy, satisfaction, application,* and *impact*. Four-step evaluation process also described: 1) *Identify project context affecting evaluation*, 2) *Identify evaluation context affecting method*, 3) *Design evaluation process based on the project and evaluation context*, and 4) *Execute evaluation plan and use learnings to improve current and future projects.*	Evaluation framework developed as part of workshop process, based on participants' understanding and supported by literature review. Participants not clearly identified, but reported to have diverse range of expertise including "…social and natural sciences, public health, and computer science [with] extensive experience in the development of models for decision and policy support, social learning, and scientific research." Built on work of Cash *et al.,* 2003,[79] Goeller (1998),[84] and Roughley (2009),[85] with note that on their own, these frameworks are too generic and only relevant to environmental modelling processes.	Total of 32 evaluation criteria presented, with demonstration of how three common types of research methods – decision support systems, PM and research modelling – should prioritize the criteria for evaluation purposes. Overview of common evaluation research methodologies presented in *Table 4.*	Levels of evaluation impacts differentiated, including *project-level* (within project timeframe), *group- and individual-level impacts* (apparent in short to long term), *system-level* (becoming apparent in long-term). Evaluation criteria and process to be applied both before (*ex ante*) and after (*ex post*) the PM program.	*Strengths.* The most comprehensive PM evaluation framework that exists to date. Though evaluation criteria are specific to environmental modelling studies, can be adapted across diverse research disciplines. *Limitations*. Though 32 evaluation criteria are very comprehensive, it is not always pragmatic and realistic to achieve in all evaluation research studies. There is no research evidence of the actual effectiveness of the evaluation framework when applied in a PM program. Research tools are also not provided to guide the adoption of the framework in evaluation practice.

Waterlander (2020)	Evaluation process designed to understand how the system evolves under influence of the LIKE programme, which aims to address the complex problem of childhood overweight and obesity in 10-14-year-old adolescents through PM.	Evaluation process built on work by Moore *et al.,* 2015;[86] Walton (2015),[87] and; Egan *et al.,* 2019.[88] Also developed underpinned by principles of Participatory Action Research and developmental evaluation.	Evaluation process will be applied in the LIKE programme employing qualitative and quantitative methods to assess changes in health behavior and body weight that result from the programme and interpret these outcomes in relation to the system changes.	Consideration of prospective evaluation data collection that measures pre-existing systems maps, with changes over time at different levels of the system elements, system structures, and/or the system as a whole; similar to that proposed by Egan *et al.* Placed specific emphasis on achieving changes at higher system levels throughout the action-programme development using the Intervention Level Framework.	*Strengths.* Developmental systems evaluation design, built on principles of Participatory Action Research, leads to adaptive evaluation process that has the potential to respond to changes at different levels of the system. *Limitations.* No research evidence currently available regarding effectiveness of the evaluation process when applied in a PM program, such as LIKE.

Zare (2021)	Formative, self-reflective monitoring and evaluation process described consisting of six steps: *1) Review of documents to understand research goals, priorities and records*, *2) Pathways assessment, 3) Reflective meetings with each team member, 4) Analysis of qualitative data from meetings to capture perceived strengths, weaknesses, success and failures, and suggested actions, objectives and goals, 5) Survey to elicit feedback on identified goals, objectives and actions,* and *6) Report of approved actions based on the conducted survey of team members.*	Monitoring and evaluation process developed as a result of the synthesis of methods from Gibbs (1988),[89] Holzer *et al.* (2018),[90] Kunseler *et al.* (2015),[91] van Mierlo *et al.* (2010),[92] Zare *et al*. (2020).[93]	Evaluation process applied to an integrated assessment of water allocation and use opportunities modelling project in the Campaspe catchment, part of the Murray-Darling Basin in Victoria (Australia), to respond to challenges regarding water availability.	Recognition that monitoring and evaluation processes are an integral activity during all steps/phases of PM to aim for ambitious outcomes and modify activities over time, as needed.	*Strengths.* Adaptive and flexible monitoring and evaluation process to suit the needs of complex problem solving. *Limitations.* A general process is described, rather a comprehensive evaluation framework/criteria. The authors have recognized that it would have been advantageous to have the formative and reflective monitoring and evaluation to be part of a complex, participatory project from the outset.

Bold and italics = clear evaluation framework and/or criteria defined, with case study described; **Bold = clear evaluation framework and/or criteria defined, with no case study***; italics = evaluation process defined, with case study described*

Overall, key benefits and areas of future research were identified for each paper through the data extraction process (i.e., imPact and Prioritizing categories of Freebairn's adapted 6P communications framework on reporting PM outcomes). For example, Lynam *et al.*'s paper was one of the first academic papers that attempted to address the gap in research evidence to support improved evaluation practices in PM.[54] It was also one of the first to identify the need to address power relations when working with communities, as well as the PM process distinct from the technical model (e.g., encouraging co-learning/communication *vs* level of accuracy/precision).[54] As such, Lynam *et al.* was referenced by various other papers,[24, 37, 55, 58, 59] and was used by Zorrilla *et al.* as a basis to develop their own evaluation framework.[55] While Lynam *et al.*'s work was ground-breaking being one of the first in this field, it was limited in providing a comprehensive description of the theoretical underpinnings of their framework.

A strength of some of the identified evaluation frameworks, criteria and/or processes was that they were generalizable enough to be applied to other PM programs.[23, 24, 59] However, for some this came at the cost of oversimplifying the evaluation framework.[24, 55, 56, 61] The strengths and limitations of each individual study are presented in Table 5. Recurring themes were synthesized from across the papers utilizing Freebairn's adapted 6P communications framework for reporting on PM programs.[13] The themes are presented in Table 6.

The ability of the framework to work not only for the specific PM program evaluated is a key criteria.

Table 6. Recurring themes applied to Freebairn's adapted six-dimensional (6P) communications framework that standardize the reporting of PM studies.

Six-dimensional reporting criteria	Synthesized evaluation themes from included studies
Purpose (e.g., why PM approaches should be evaluated)	To develop an evaluation framework and/or criteria for PM programs and/or tools.<br><br>The aim differed across studies, but ranged from evaluating the: success of PM programs with consideration of participatory processes;[23, 24, 37, 58, 61]

	changes in systems behavior to address complex challenges;[60] changes in stakeholder behaviors such as learning, decision making and values;[57, 59] PM tools,[54, 55] and; difference between modelling outcomes and outputs.[56]
Process (e.g., method utilized to execute evaluation framework/criteria/process)	Not described in all studies. Various* evaluation methods described including: questionnaires;[23, 55, 57, 61] *ex post* evaluation;[23, 24, 57] *ex ante* evaluation;[23, 57] process evaluation;[24, 55, 58] evaluation forms;[56] workshop recordings;[56, 57, 60] semi-structured interviews;[23, 37, 55, 58, 60, 93] researchers' theoretical assessment;[55, 60] social network analysis;[60] participant observation;[23, 60] focus groups;[23] pre and post testing;[23] document analysis;[23] informal conversational meetings;[23] formative evaluation,[23, 61] and outcome evaluation.[56]
Partnerships (e.g., stakeholders involved in the development of the evaluation framework/criteria/process)	Not described in all studies. Mention of discussion-based process amongst authors,[24, 54] and; workshop process with stakeholders.[23]
Products (e.g., level of evaluation – theoretical, conceptual, implementation)	Theoretical evaluation framework and/or criteria only with no presented application case study;[23, 54] theoretical evaluation framework and/or criteria presented with case study described;[24, 37, 55, 58, 59] and; evaluation framework described as a process described with case study described.[56, 57, 60, 61]
imPact (e.g., outcomes/strengths of the evaluation framework/criteria/process)	Various* outcomes identified including: flexible framework to be applied to other disciplines;[23, 54, 59, 61] consideration of participatory process as well as influence of actual technical model;[23, 37, 55] supports decision making;[23, 24, 54] evaluation considers the whole process that can be adapted for each unique study's context and purpose;[23] evaluation of interventions in complex settings;[60] prompts action and reflection in evaluation design to cater to dynamic nature of complex systems;[60, 61] evaluate the engagement, learning and/or integration of stakeholders in PM;[55, 57, 59] evaluation of PM outcomes and outputs,[23, 37, 55, 56] and; consideration of short- and long term outcomes.[23, 58]
Prioritizing (e.g., barriers, future opportunities of the evaluation framework/criteria/process)	Various* barriers/future opportunities identified including: lack of empirical evidence regarding the development of the presented evaluation framework;[54, 57] evaluation framework, criteria and/or process arguably oversimplified;[24, 55, 56, 61] evaluation framework development and/or application not discussed in depth;[37, 58, 60] evaluation focuses solely on the participatory process without factoring how the participatory process influences the model implementation;[59] and; no demonstrated

	effectiveness of the evaluation framework through case study.[23, 60]

**Some studies may be mentioned more than once, as they have employed more than one approach.*

Challenges and risks identified

It was evident from the synthesis process that differing terminologies, approaches, and assumptions were used, which led to challenges in determining the best evaluation framework to adopt. This poses risk that evaluation processes will not reach their full potential, which has implications for funders, participating stakeholders, as well as modellers.[94] Additionally, it was evident during the synthesis process that studies either described a comprehensive evaluation framework, criteria and/or process, or they focused on the actual evaluation methodologies; the two rarely coincided.[95, 96] Therefore, a comprehensive evaluation framework and criteria are needed for PM programs that have theoretical and empirical underpinnings but are also accompanied by practical evaluation tools and methods to support real-world implementation.

Scope of synthesis and development of comprehensive multi-scale evaluation framework

To analyze the heterogeneity in terminology identified during the data extraction process for the studies included, a word cloud was generated aligned to Hearst *et al.*'s recommendations on developing effective word clouds to improve reader understanding (Figure 2).[97] Word clouds visually display the most frequently used words in a body of text; the bigger font size of a word illustrates that this word is used more frequently.[98] To ensure that a focused word cloud was generated specific to evaluation, the authors first uploaded the full text of all 11 studies included for synthesis. A process of elimination was performed, whereby words that were not related to evaluation – such as *university, platform,* and various stop words – were deleted. Following this process, the authors went through the remaining list of words and merged synonyms as well as the same words presented in its singular or plural form or with tense variation.

There is a problem with the idea to find or define the ”best” evaluation framework. See comment above.

See Hedelin et al. (2021) for such an attempt.

[Suggested location of figure]

Figure 2. Word cloud generated by uploading the full text of all studies included for synthesis.

There are limitations to developing a word cloud. For example, it is not clear from just the word cloud exercise alone how many terms appeared in each individual study. However, through this exercise, the authors identified a total of 40 unique evaluation terms that were most used across the studies included for synthesis. *Process* appeared the most frequently (623 times), whereas *inclusive* appeared the least (six times). The final list of terms was further analyzed and incorporated into the comprehensive PM evaluation framework. Most of the terms identified fit into four categories – *feasibility, value, change & action (impact)* and *sustainability*, Thus, these four terms were identified as the evaluation framework categories (highlighted in yellow, Table 7). The remaining 35 terms (highlighted in grey, Table 7) have been incorporated into the evaluation criteria, or evaluation questions. The word *level* was neither incorporated as an evaluation framework category nor criteria; but as the various levels of evaluation (e.g., project-level *vs* system-level) were noteworthy across studies, this term was included as a separate component in our evaluation framework (Table 8).

Define level of evaluation early on in the text (before table 5), and not just by an example.

Table 7. Words extracted for development of multi-scale evaluation framework.

The concept Sustainable often have very different meanings than the ”sustainable over time” meaning you give it with in your framework. PM is common in environmental planning/ management settings, where sustainable mainly means how resources are used and distributed etc. The word count/ cloud does not differ between these very different meanings…

Term	# of times the term appeared across studies
process	623
decision/decision making	461
change	333
outcome	304
action	301
value	212
context	181
level	175
reflection	170
policy	164
effective	157
learn	141
engage	138
time	106
impact	102
network	98
communication	95
planning	92
resource	89

behavior	89
sustainable	82
feasible	78
relationship	67
belief	52
trust	52
capacity	51
needs	51
accessibility	50
credibility	50
transparency	47
feedback	36
flexibility	36
efficiency	32
simple	28
validation	28
acceptance	26
salience	26
utility	26
accuracy	8
inclusive	6

Yellow highlight suggests common themes that have formed the evaluation framework categories; grey highlight suggests common sub-themes that have formed the evaluation criteria; green highlight suggests the consideration of multiple evaluation levels to be embedded within the evaluation framework.

Discussion

Parts of the discussion feels like a result - the description of your evaluation framework. E.g. the meaning of the categories and how the words are used to formulate criteria (?)

This scoping review identified 11 studies that described an evaluation framework, criteria and/or process developed for PM programs. From the synthesis of these papers, the strengths and limitations, as well as overlapping concepts and themes were synthesized to present a comprehensive multi-scale evaluation framework (Table 8) that can be utilized by PM programs, regardless of the discipline of study or modelling method. From the process of developing a word cloud, the authors identified common terms utilized across previous evaluation frameworks, and supplemented this information with the extensive experience of the authors (IH, JO, AS, LF) in conducting PM to develop an evaluation framework that consists of four categories – (i) *Feasibility;* (ii) *Value;* (iii) *Change & Action (Impact)*, and; (iv) *Sustainability*. It is recommended that comprehensive evaluation processes need clear criteria to set appropriate benchmarks;[55, 71] therefore, the authors developed 25 criteria, which include all key words identified from the word cloud (Table 8).

Describe this in the method section…

Where are the criteria? You mean the questions?

As an evaluation concept, the *feasibilit*y or plausibility of PM allows for questions to be asked regarding whether it was possible for all participants to engage and contribute throughout the PM process. Consideration of the *value* of the PM process allows for the exploration of questions regarding what was gained due to engaging participants in PM (e.g., learning, confidence, trust). *Change & action* facilitates observations of impact, including *ex ante* and *ex post* comparisons of stakeholder relationships, knowledge, and behaviors as a result of the PM process; *sustainability* allows for the observations of these impacts over time (Table 8).

Hamilton *et al.* presented the most comprehensive evaluation framework available to date, developed through the recognition that many evaluation practices have been inadequate in both depth and scope which has limited the ability to improve PM practices.[23] Though the authors have developed the presented evaluation framework through the synthesis of other evaluation frameworks, the work presented by Hamilton *et al.* was most thoroughly studied, adapted and incorporated. For example, the proposal by Hamilton *et al.* to consider differing levels of impact from the project-, individual-, group- to system-level, was also embedded into our framework. Though Hamilton *et al.* identified that there are sublevels that may exist within the individual- and group-levels (i.e., client *vs* decision makers), this was not made explicit in their evaluation framework. Jones attempted to do this by separating out evaluation methods for the project team and the stakeholder group,[24] and we further propose examination across stakeholder groups who may participate in the PM process (e.g., workshops, meetings, etc). This is critical as PM processes are inclusive and involve stakeholders from diverse backgrounds. Consideration of the sublevels of participation enables the recognition of, for example, potential power relations and dynamics amongst the stakeholders, to be able to improve PM design and appropriately measure outcomes.[23, 24, 54-56, 59] This has been reflected in our evaluation framework, presented in Table 8, with the

individual- and group-levels further stratified to include community participants (e.g., consumer representatives) and professional participants (e.g., policy makers).

Table 8. Comprehensive multi-scale evaluation framework for PM programs.

	**FEASIBILITY**<br>***Is PM feasible?***	**VALUE**<br>***What is the value of the PM process?***	**CHANGE & ACTION (IMPACT)**<br>***What changed as a result of PM?***	**SUSTAINABILITY**<br>***Are the changes and actions sustained over time?***
**PROJECT-LEVEL IMPACT**	Is it feasible to develop systems models through participatory methods?	How did the PM process add value (e.g., context, validity, learning) to developing the systems models?	How was feedback considered throughout the program to improve the PM process (including the build of the systems model)?<br><br>Was the PM process flexible enough to take action/respond to the changing needs of the complex system?	How does the PM process promote sustained use of the systems model?
**INDIVIDUAL-LEVEL IMPACT**				
**Community Participants (e.g., consumer representatives)**	How do community participants view the credibility of the PM process?	What are the experiences arising from the application of PM to community participants (e.g., outcomes, salience, ability to share their story)?	Are there changes in perceived knowledge, beliefs, behaviors, or assumptions for participants?<br><br>Are there changes in the way participants engage with the system (e.g., reflection)?	Are there sustained changes in knowledge, beliefs, behaviors and/or assumptions for participants?
**Professional Participants (e.g., policy makers)**	How do professional participants view the credibility of the PM process?<br><br>How do professional participants view the credibility of the evidence used to effectively inform the systems model?	What are the experiences of professional participants using the systems model (e.g., confidence using the tool, ease/simple to use, salience, acceptance)?		
**GROUP-LEVEL IMPACT**				
**Community Participants**	How did the community participants contribute and engage during the PM process?	What are the experiences (e.g., benefits and challenges) working in collaboration with professional	Are there changes in social network connections and interdisciplinary	Are changes in social network connections and interdisciplinary

**(e.g., consumer groups)**		participants during PM (e.g., communication, relationships, trust, social networks)?	collaboration as a result of the PM process?	collaborations sustained over time?
**Professional Participants (e.g., policy makers)**	How were power relationships managed?<br><br>How did professional participants ensure that PM processes were inclusive, accessible, and transparent?	What are the experiences (e.g., benefits and challenges) working in interdisciplinary collaboration with community participants and/or other professional participants during the PM process (e.g., communication, relationships, trust, social networks)?		
**SYSTEM-LEVEL IMPACT** 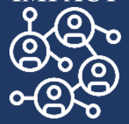	Can systems models be built through a participatory approach that can effectively inform policy, planning, and investment decisions with a degree of confidence in accuracy to address complex systems challenges?	Does the participatory approach in building systems models add sufficient value to warrant the time and resources investment (e.g., improve capacity/efficiency, confidence)?	How have insights from the PM process been applied in the complex system of interest?<br><br>What are the factors that have influenced the extent to which the systems model has been utilized?	How have insights from the systems models been applied in the longer term?<br><br>How do participants' engagement with and use of the systems model change over time?<br><br>What are the longer-term factors that have influenced the extent to which the systems model is ongoingly utilized to inform policy, planning, and investment decisions?

The principles of Participatory Action Research (PAR) underpin the proposed evaluation framework (Figure 3). PAR aims to improve outcomes and reduce inequities by working with the people who systems model most affects, such as consumer representatives.[99] The PAR approach is appropriate in the context of PM as the traditional roles of the modellers as the experts and stakeholders as the study participants are challenged.[100, 101] In the PM process stakeholders are invited to contribute their expertise and experience about their understanding of the system of interest, making both participatory modellers and stakeholders equal and active participants.[99] PAR also embeds reflection during all phases of the PM program and can lead to shared learning and joint action for change to improve PM processes.[21, 22]

[Suggested location of figure]

Figure 3. Drawing on the principles of participatory action research, the comprehensive multi-scale evaluation framework should be executed with consideration of impacts across the project-, individual-, group, and system-level.

The studies included for synthesis (Table 5, Table 6) used a variety of methods to collect evaluation data. It is recommended that the presented evaluation framework adopts a mixed methods approach to align with the PM process. Examples of the potential methods include semi-structured interviews, surveys, journey maps and social network analysis (Figure 4). A more thorough description of how the presented evaluation framework can be executed through a mixed-methods approach, including the tools the authors have developed as well as the suggested evaluation time points (*ex ante* and *ex post*), is described elsewhere in the context of youth mental health participatory systems modelling.

Make a reference to this text.

[Suggested location of figure]

Figure 4. Methods of data collection to adopt the comprehensive multi-scale framework into evaluation practice.

Evaluation can do many other things as well. E.g. provide understanding of the process and what parts of it that has a certain effect. So that the knowledge can be transferred more easily since every process is dependent on context. Before-after-measurements will not be so helpful in that sense.

Strengths, limitations and opportunities for future research

Evaluations have the potential to measure change at the project, individual, group, and system (policy) level.[23, 102, 103] Careful thought on design aspects are needed to ensure that evaluations are worthwhile as they require additional time, resources, and funding.[104] The presented evaluation framework considers the contributions of all participants involved in the PM process, not only the perspectives of the modellers or funder.[104, 105]

The presented evaluation framework is also designed to be adaptive, flexible and iterative, to ensure that the framework remains relevant despite the evolving field and contexts in which PM are being applied.[9] The proposed evaluation framework builds on principles of PAR to empower stakeholders from various backgrounds (e.g., community participants to professional participants), and embeds ongoing reflection and learning so that the PM process can respond to the changing needs of complex systems, as well as be applied across disciplines and diverse modelling methods.[21] The presented evaluation framework supports the application of a mixed-methods approach with an emphasis on approaching PM evaluations holistically.[106]

Yes, but the description of how these principles are translated into evaluation activities, more practically, is mainly lacking.

There are limitations to this scoping review that should be acknowledged. The heterogeneity in terminology was a challenge during the screening, data extraction, and full text review process. However, with the process described in which the first author and senior author worked closely to resolve any ambiguity, a robust method was followed to ensure that the studies included are most relevant for the purposes of this scoping review. Additionally, though the described search strategy was broad in that it did not set any limits to the field of study, it was narrow so that only the studies that disclosed an evaluation framework, criteria and/or process in a PM context were included.

Conclusions

Evaluations are an integral component of the PM process that should be carefully considered throughout, and not viewed as its own separate component or afterthought. With the ability to inform policy change by demonstrating the measured effectiveness of PM, such processes should be adequately supported with an appropriate evaluation design. The presented framework describes a multi-scale and comprehensive, yet flexible evaluation approach that is built on the rigorous synthesis of strengths and opportunities for further development identified from existing studies. This framework enables the conduct of holistic evaluation practices by considering the project-, individual-, group-, and system-level impacts to understand the feasibility, value, impact, and sustainability of the PM process. Outputs from adopting such an evaluation approach, underpinned by principles of PAR, can be used to guide ongoing improvements to the PM process, empower stakeholders and users of systems models to be more confident in the model outcomes, as well as to improve understanding of which aspects of PM are particularly important for policy decisions.

Acknowledgements

The authors would like to thank Glen Smyth, Academic Liaison Librarian at The University of Sydney, for his guidance and support in developing the search strategy. The authors would also like to thank Chloe Wilson for her support in assisting with the initial literature search.

References

1. Bala BK. AF, Noh KM. System Dynamics: Modelling and Simulation. Singapore Springer Nature 2017.
2. Kelly RA, Jakeman AJ, Barreteau O, Borsuk ME, ElSawah S, Hamilton SH, et al. Selecting among five common modelling approaches for integrated environmental assessment and management. Environmental Modelling & Software. 2013;47:159-81. doi: https://doi.org/10.1016/j.envsoft.2013.05.005.
3. Borschev A, Grigoryev I. The Big Book of Simulation Modeling: Multimethod Modeling with AnyLogic 6. Chicago: AnyLogic North America 2013.

4. Currie DJ, Smith C, Jagals P. The application of system dynamics modelling to environmental health decision-making and policy - a scoping review. BMC Public Health. 2018;18(1):402. doi: 10.1186/s12889-018-5318-8.

5. Freebairn L, Atkinson J, Kelly PM, McDonnell G, Rychetnik L. Decision makers' experience of participatory dynamic simulation modelling: methods for public health policy. BMC Medical Informatics and Decision Making. 2018;18(1):131. doi: 10.1186/s12911-018-0707-6.

6. Hieronymi A. Understanding Systems Science: A Visual and Integrative Approach. Systems Research and Behavioral Science. 2013;30(5):580-95. doi: https://doi.org/10.1002/sres.2215.

7. Long KM, Meadows GN. Simulation modelling in mental health: A systematic review. Journal of Simulation. 2018;12(1):76-85. doi: 10.1057/s41273-017-0062-0.

8. Luke DA, Stamatakis KA. Systems science methods in public health: dynamics, networks, and agents. Annu Rev Public Health. 2012;33:357-76. Epub 2012/01/03. doi: 10.1146/annurev-publhealth-031210-101222. PubMed PMID: 22224885.

9. Prell C, Hubacek K, Reed M, Quinn C, Jin N, Holden J, et al. If you have a hammer everything looks like a nail: traditional versus participatory model building. Interdisciplinary Science Reviews. 2007;32(3):263-82. doi: 10.1179/030801807X211720.

10. Pitt M, Monks T, Crowe S, Vasilakis C. Systems modelling and simulation in health service design, delivery and decision making. BMJ Quality & Safety. 2016;25(1):38. doi: 10.1136/bmjqs-2015-004430.

11. Osgood N. Computational Simulation Modeling in Population Health Research and Policy. In: Apostolopoulos Y. LK, Lemke MK, editor. Complex Systems and Population Health. New York Oxford University Press 2020.

12. Silva PCL, Batista PVC, Lima HS, Alves MA, Guimarães FG, Silva RCP. COVID-ABS: An agent-based model of COVID-19 epidemic to simulate health and economic effects of social distancing interventions. Chaos Solitons Fractals. 2020;139:110088-. Epub 2020/07/07. doi: 10.1016/j.chaos.2020.110088. PubMed PMID: 32834624.

13. Freebairn L. "Turning mirrors into windows": A study of participatory dynamic simulation modelling to inform health policy decisions: The University of Notre Dame Australia 2019.

14. Rutter H, Savona N, Glonti K, Bibby J, Cummins S, Finegood DT, et al. The need for a complex systems model of evidence for public health. The Lancet. 2017;390(10112):2602-4. doi: 10.1016/S0140-6736(17)31267-9.

15. Kendall JM. Designing a research project: randomised controlled trials and their principles. Emergency Medicine Journal. 2003;20(2):164. doi: 10.1136/emj.20.2.164.

16. Page A, Atkinson JA, Heffernan M, McDonnell G, Prodan A, Osgood N, et al. Static metrics of impact for a dynamic problem: The need for smarter tools to guide suicide prevention planning and investment. Aust N Z J Psychiatry. 2018;52(7):660-7. Epub 2018/01/24. doi: 10.1177/0004867417752866. PubMed PMID: 29359569.

17. Atkinson J, Skinner A, Lawson K, Rosenberg S, Hickie IB. Bringing new tools, a regional focus, resource-sensitivity, local engagement and necessary discipline to mental health policy and planning. BMC Public Health. 2020;20(1):814. doi: 10.1186/s12889-020-08948-3.

18. Occhipinti J, Skinner A, Carter S, Heath J, Lawson K, McGill K, et al. Federal and state cooperation necessary but not sufficient for effective regional mental health systems: insights from systems modelling and simulation. Scientific Reports. 2021;11(1):11209. doi: 10.1038/s41598-021-90762-x.

19. Page A, Atkinson J, Campos W, Heffernan M, Ferdousi S, Power A, et al. A decision support tool to inform local suicide prevention activity in Greater Western Sydney

(Australia). Aust N Z J Psychiatry. 2018;52(10):983-93. Epub 2018/04/20. doi: 10.1177/0004867418767315. PubMed PMID: 29671335.
20. Senge PM, Sterman JD. Systems thinking and organizational learning: Acting locally and thinking globally in the organization of the future. European Journal of Operational Research. 1992;59(1):137-50. doi: https://doi.org/10.1016/0377-2217(92)90011-W.
21. Voinov A, Bousquet F. Modelling with stakeholders. Environmental Modelling & Software. 2010;25(11):1268-81. doi: https://doi.org/10.1016/j.envsoft.2010.03.007.
22. Gilbert N, Ahrweiler P, Barbrook-Johnson P, Narasimhan KP, Wilkinson H. Computational Modelling of Public Policy: Reflections on Practice. Journal of Artificial Societies and Social Simulation. 2018;21(1):14. doi: 10.18564/jasss.3669.
23. Hamilton SH, Fu B, Guillaume JHA, Badham J, Elsawah S, Gober P, et al. A framework for characterising and evaluating the effectiveness of environmental modelling. Environmental Modelling & Software. 2019;118:83-98. doi: https://doi.org/10.1016/j.envsoft.2019.04.008.
24. Jones NA, Perez P, Measham TG, Kelly GJ, d'Aquino P, Daniell KA, et al. Evaluating Participatory Modeling: Developing a Framework for Cross-Case Analysis. Environmental Management. 2009;44(6):1180. doi: 10.1007/s00267-009-9391-8.
25. Király G, Miskolczi P. Dynamics of participation: System dynamics and participation—An empirical review. Systems Research and Behavioral Science. 2019;36(2):199-210. doi: https://doi.org/10.1002/sres.2580.
26. Liguori A, McEwen L, Blake J, Wilson M. Towards 'Creative Participatory Science': Exploring Future Scenarios Through Specialist Drought Science and Community Storytelling. Frontiers in Environmental Science. 2021;8(293). doi: 10.3389/fenvs.2020.589856.
27. Maru YT. AK, Perez P. Taking 'participatory' in participatory modelling seriously 18th World IMAC/MODSIM Congress; Cairns2009.
28. Hovmand P. Community Based System Dynamics. New York Springer 2013.
29. Voinov A, Jenni K, Gray S, Kolagani N, Glynn PD, Bommel P, et al. Tools and methods in participatory modeling: Selecting the right tool for the job. Environmental Modelling & Software. 2018;109:232-55. doi: https://doi.org/10.1016/j.envsoft.2018.08.028.
30. Gates EF. Making sense of the emerging conversation in evaluation about systems thinking and complexity science. Evaluation and Program Planning. 2016;59:62-73. doi: https://doi.org/10.1016/j.evalprogplan.2016.08.004.
31. Gopalakrishnan S PH, Lu SJ. Next Generation Evaluation: Embracing Complexity, Connectivity, and Change: A Learning Brief. Boston, MA: 2013.
32. Preskill H. GS, Mack K., Cook J. Evaluating Complexity Propositions for Improving Practice Boston: 2014.
33. Centers for Disease Control and Prevention. Types of Evaluation Atlanta Centers for Disease Control and Prevention, n.d.
34. Carpenter J. 7 - Evaluation and review. In: Carpenter J, editor. Project Management in Libraries, Archives and Museums: Chandos Publishing; 2011. p. 127-49.
35. Worthen BR. "Beyond Formative and Summative Evaluation" Revisited. Evaluation Practice. 1996;17(2):119-. doi: 10.1177/109821409601700203.
36. Program Evaluation. 1999 2021/07/07. London: SAGE Publications, Ltd. Available from: https://methods.sagepub.com/book/program-evaluation.
37. Falconi SM, Palmer RN. An interdisciplinary framework for participatory modeling design and evaluation—What makes models effective participatory decision tools? Water Resources Research. 2017;53(2):1625-45. doi: https://doi.org/10.1002/2016WR019373.

38. Gertler PJ, Martinez S, Premand P, Rawlings LB, Vermeersch CMJ. Impact Evaluation in Practice, Second Edition. Washington, D. C., UNITED STATES: World Bank Publications; 2016.
39. The Department of Health. Why evaluate? Canberra Australian Government 2012. Available from: https://www1.health.gov.au/internet/publications/publishing.nsf/Content/evaluation-tkit-breastfeeding-prgms-projects~evaluate.
40. Cambridge Dictionary. Meaning of "evaluation" in English: Cambridge Dictionary; n.d. Available from: https://dictionary.cambridge.org/dictionary/english/evaluation.
41. Fynn JF, Hardeman W, Milton K, Murphy J, Jones A. A systematic review of the use and reporting of evaluation frameworks within evaluations of physical activity interventions. International Journal of Behavioral Nutrition and Physical Activity. 2020;17(1):107. doi: 10.1186/s12966-020-01013-7.
42. Gray S, Voinov A, Paolisso M, Jordan R, BenDor T, Bommel P, et al. Purpose, processes, partnerships, and products: four Ps to advance participatory socio-environmental modeling. Ecol Appl. 2018;28(1):46-61. Epub 2017/09/19. doi: 10.1002/eap.1627. PubMed PMID: 28922513.
43. Forss K, Marra M, Schwartz R. Evaluating the Complex: Attribution Contribution and Beyond. New Burnswick (USA) and London (UK): Transaction Publishers; 2011.
44. Jordan R, Gray S, Zellner M, Glynn PD, Voinov A, Hedelin B, et al. Twelve Questions for the Participatory Modeling Community. Earth's Future. 2018;6(8):1046-57. doi: https://doi.org/10.1029/2018EF000841.
45. Hassenforder E, Pittock J, Barreteau O, Daniell KA, Ferrand N. The MEPPP Framework: A Framework for Monitoring and Evaluating Participatory Planning Processes. Environmental Management. 2016;57(1):79-96. doi: 10.1007/s00267-015-0599-5.
46. Rychetnik L, Frommer M, Hawe P, Shiell A. Criteria for evaluating evidence on public health interventions. J Epidemiol Community Health. 2002;56(2):119-27. Epub 2002/01/29. doi: 10.1136/jech.56.2.119. PubMed PMID: 11812811; PubMed Central PMCID: PMCPMC1732065.
47. Munn Z, Peters MDJ, Stern C, Tufanaru C, McArthur A, Aromataris E. Systematic review or scoping review? Guidance for authors when choosing between a systematic or scoping review approach. BMC Medical Research Methodology. 2018;18(1):143. doi: 10.1186/s12874-018-0611-x.
48. Pham MT, Rajić A, Greig JD, Sargeant JM, Papadopoulos A, McEwen SA. A scoping review of scoping reviews: advancing the approach and enhancing the consistency. Res Synth Methods. 2014;5(4):371-85. Epub 2014/07/24. doi: 10.1002/jrsm.1123. PubMed PMID: 26052958.
49. Peters MDJ GC, McInerney P, Munn Z, Tricco AC, Khalil H. Chapter 11: Scoping Reviews: JBI; 2020. Available from: https://synthesismanual.jbi.global. https://doi.org/10.46658/JBIMES-20-12.
50. Arksey H, O'Malley L. Scoping studies: towards a methodological framework. International Journal of Social Research Methodology. 2005;8(1):19-32. doi: 10.1080/1364557032000119616.
51. Moher D, Liberati A, Tetzlaff J, Altman DG. Preferred reporting items for systematic reviews and meta-analyses: the PRISMA statement. BMJ. 2009;339:b2535. doi: 10.1136/bmj.b2535.
52. Registration of scoping review: Open Science Framework; 2021. Available from: https://osf.io/9dxu8/?view_only=.
53. Alberta Addiction and Mental Health Research Partnership Program. Evaluation Plan and Evaluation Framework. Alberta: Alberta Health Services; n.d.

54. Lynam T. dJW, Sheil D., Kusumanto T., Evans K. A Review of Tools for Incorporating Community Knowledge, Preferences, and Values into Decision Making in Natural Resources Management. Ecology and Society 2007;12:5.

55. Zorilla-Miras P. CG, Hera A., Varela-Ortega, Martinez-Santos P, Bromley J, Henriksen H. Evaluation of Bayesian Networks in Participatory Water Resources Management, Upper Guadiana Basin, Spain. Ecology and Society 2010;15(3). doi: 10.5751/ES-03278-150312.

56. Matthews KB, Rivington M, Blackstock K, McCrum G, Buchan K, Miller DG. Raising the bar? – The challenges of evaluating the outcomes of environmental modelling and software. Environmental Modelling & Software. 2011;26(3):247-57. doi: https://doi.org/10.1016/j.envsoft.2010.03.031.

57. Smajgl A, Ward J. Evaluating participatory research: Framework, methods and implementation results. J Environ Manage. 2015;157:311-9. Epub 2015/05/02. doi: 10.1016/j.jenvman.2015.04.014. PubMed PMID: 25929196.

58. Maskrey SA, Mount NJ, Thorne CR, Dryden I. Participatory modelling for stakeholder involvement in the development of flood risk management intervention options. Environmental Modelling & Software. 2016;82:275-94. doi: https://doi.org/10.1016/j.envsoft.2016.04.027.

59. Hedelin B, Evers M, Alkan-Olsson J, Jonsson A. Participatory modelling for sustainable development: Key issues derived from five cases of natural resource and disaster risk management. Environmental Science & Policy. 2017;76:185-96. doi: https://doi.org/10.1016/j.envsci.2017.07.001.

60. Waterlander WE, Luna Pinzon A, Verhoeff A, den Hertog K, Altenburg T, Dijkstra C, et al. A System Dynamics and Participatory Action Research Approach to Promote Healthy Living and a Healthy Weight among 10-14-Year-Old Adolescents in Amsterdam: The LIKE Programme. Int J Environ Res Public Health. 2020;17(14):4928. doi: 10.3390/ijerph17144928. PubMed PMID: 32650571.

61. Zare F, Guillaume JHA, ElSawah S, Croke B, Fu B, Iwanaga T, et al. A formative and self-reflective approach to monitoring and evaluation of interdisciplinary team research: An integrated water resource modelling application in Australia. Journal of Hydrology. 2021;596:126070. doi: https://doi.org/10.1016/j.jhydrol.2021.126070.

62. Argyris C. On Organizational Learning. 2nd Edition ed. New Jersey Wiley-Blackwell; 1999.

63. Patton M. Qualitative evaluation and research methods. Newbury Park, California: Sage Publications; 1992.

64. Curnan S LL, Sharpstee D, Lelle M, Reece M. W.K Kellogg Foundation evaluation handbook. Battle Creek, Michigan, United States: 1998.

65. Abelson J, Forest P-G, Eyles J, Smith P, Martin E, Gauvin F-P. Deliberations about deliberative methods: issues in the design and evaluation of public participation processes. Social Science & Medicine. 2003;57(2):239-51. doi: https://doi.org/10.1016/S0277-9536(02)00343-X.

66. Rowe G, Frewer LJ. Evaluating Public-Participation Exercises: A Research Agenda. Science, Technology, & Human Values. 2004;29(4):512-56. doi: 10.1177/0162243903259197.

67. Korff YV, editor Towards an Evaluation Method for Public Participation Processes in AquaStress and NeWater A proposal for both projects2005.

68. Stewart TR, Dennis RL, Ely DW. Citizen participation and judgment in policy analysis: A case study of urban air quality policy. Policy Sciences. 1984;17(1):67-87. doi: 10.1007/BF00229729.

69. Breck EFEEJ. Public Understanding of Science. 2001;10(1):83-98. doi: 10.1088/0963-6625/10/1/306.
70. Henriksen HJ, Rasmussen P, Brandt G, von Bülow D, Jensen FV. Public participation modelling using Bayesian networks in management of groundwater contamination. Environmental Modelling & Software. 2007;22(8):1101-13. doi: https://doi.org/10.1016/j.envsoft.2006.01.008.
71. Blackstock KL, Kelly GJ, Horsey BL. Developing and applying a framework to evaluate participatory research for sustainability. Ecological Economics. 2007;60(4):726-42. doi: https://doi.org/10.1016/j.ecolecon.2006.05.014.
72. Patton M. Utilization-Focused Evaluation. In: Kellaghan T. SD, editor. International Handbook of Educational Evaluation. 9. Dordrecht: Springer; 2003.
73. McCown RL. Changing systems for supporting farmers' decisions: problems, paradigms, and prospects. Agricultural Systems. 2002;74(1):179-220. doi: https://doi.org/10.1016/S0308-521X(02)00026-4.
74. Smajgl A, Ward J. A framework to bridge science and policy in complex decision making arenas. Futures. 2013;52:52-8. doi: https://doi.org/10.1016/j.futures.2013.07.002.
75. Beierle TC. Using social goals to evaluate public participation in environmental decisions. Review of Policy Research. 1999;16(3-4):75-103. doi: https://doi.org/10.1111/j.1541-1338.1999.tb00879.x.
76. Rowe G, Frewer LJ. Public Participation Methods: A Framework for Evaluation. Science, Technology, & Human Values. 2000;25(1):3-29. doi: 10.1177/016224390002500101.
77. Webler T, Tuler S. Unlocking the Puzzle of Public Participation. Bulletin of Science, Technology & Society. 2002;22(3):179-89. doi: 10.1177/02767602022003002.
78. Council NR. Public Participation in Environmental Assessment and Decision Making. Dietz T, Stern PC, editors. Washington, DC: The National Academies Press; 2008. 322 p.
79. Cash DW, Clark WC, Alcock F, Dickson NM, Eckley N, Guston DH, et al. Knowledge systems for sustainable development. Proceedings of the National Academy of Sciences. 2003;100(14):8086. doi: 10.1073/pnas.1231332100.
80. Carr G, Blöschl G, Loucks DP. Evaluating participation in water resource management: A review. Water Resources Research. 2012;48(11). doi: https://doi.org/10.1029/2011WR011662.
81. Hedelin B. Criteria for the assessment of sustainable water management. Environ Manage. 2007;39(2):151-63. Epub 2006/12/13. doi: 10.1007/s00267-004-0387-0. PubMed PMID: 17160512.
82. Hedelin B. Further development of a sustainable procedure framework for strategic natural resources and disaster risk management. Journal of Natural Resources Policy Research. 2015;7(4):247-66. doi: 10.1080/19390459.2015.1015815.
83. Hedelin B. The Sustainable Procedure Framework for Disaster Risk Management: Illustrated by the Case of the EU Floods Directive in Sweden. International Journal of Disaster Risk Science. 2016;7(2):151-62. doi: 10.1007/s13753-016-0093-6.
84. Goeller B. A framework for evaluating success in systems analysis. In: Miser H, Quade, ES, editor. Handbook of system analysis: craft issues and procedural choices: John Wiley & Sons Ltd; 1988. p. 568-618.
85. Roughley A. Developing and using program logic in natural resource management: user guide. 2009.
86. Moore GF, Audrey S, Barker M, Bond L, Bonell C, Hardeman W, et al. Process evaluation of complex interventions: Medical Research Council guidance. Bmj. 2015;350:h1258. Epub 2015/03/21. doi: 10.1136/bmj.h1258. PubMed PMID: 25791983;

PubMed Central PMCID: PMCPMC4366184 of interests and have no relevant interests to declare. Provenance and peer review: Not commissioned; externally peer reviewed.
87. Walton M. Applying complexity theory: A review to inform evaluation design. Evaluation and Program Planning. 2014;45:119-26. doi: https://doi.org/10.1016/j.evalprogplan.2014.04.002.
88. Egan M, McGill, E., Penney, T., Anderson de Cuevas, R., Er, V., Orton, L., White, M., Lock, K., Cummins, S., Savona, N., et al. Guidance on Systems Approaches to Local Public Health Evaluation. London, UK: 2019.
89. Gibbs G. Learning By Doing: Oxford Brooks University 1988.
90. Holzer JM, Carmon N, Orenstein DE. A methodology for evaluating transdisciplinary research on coupled socio-ecological systems. Ecological Indicators. 2018;85:808-19. doi: https://doi.org/10.1016/j.ecolind.2017.10.074.
91. Kunseler E-M, Tuinstra W, Vasileiadou E, Petersen AC. The reflective futures practitioner: Balancing salience, credibility and legitimacy in generating foresight knowledge with stakeholders. Futures. 2015;66:1-12. doi: https://doi.org/10.1016/j.futures.2014.10.006.
92. van Mierlo B, Arkesteijn M, Leeuwis C. Enhancing the Reflexivity of System Innovation Projects With System Analyses. American Journal of Evaluation. 2010;31(2):143-61. doi: 10.1177/1098214010366046.
93. Zare F, Guillaume JHA, Jakeman AJ, Torabi O. Reflective communication to improve problem-solving pathways: Key issues illustrated for an integrated environmental modelling case study. Environmental Modelling & Software. 2020;126:104645. doi: https://doi.org/10.1016/j.envsoft.2020.104645.
94. O'Sullivan RG. Collaborative Evaluation within a framework of stakeholder-oriented evaluation approaches. Evaluation and Program Planning. 2012;35(4):518-22. doi: https://doi.org/10.1016/j.evalprogplan.2011.12.005.
95. Louder E, Wyborn C, Cvitanovic C, Bednarek AT. A synthesis of the frameworks available to guide evaluations of research impact at the interface of environmental science, policy and practice. Environmental Science & Policy. 2021;116:258-65. doi: https://doi.org/10.1016/j.envsci.2020.12.006.
96. Cabrera D, Trochim WMK. A protocol of systems evaluation. Ithaca, NY: 2006.
97. Hearst MA, Pedersen E, Patil L, Lee E, Laskowski P, Franconeri S. An Evaluation of Semantically Grouped Word Cloud Designs. IEEE Transactions on Visualization and Computer Graphics. 2020;26(9):2748-61. doi: 10.1109/TVCG.2019.2904683.
98. American Institutes for Research. Effective Use of Word Clouds. Arlington, VA: Model Systems Knowledge Translation Center; n.d.
99. Baum F, MacDougall C, Smith D. Participatory action research. Journal of Epidemiology and Community Health. 2006;60(10):854. doi: 10.1136/jech.2004.028662.
100. Pain R. WG, Milledge D., Lune Rivers Trust Participatory Action Research Toolkit Durham, UK 2011.
101. Rodríguez LF, Brown TM. From voice to agency: guiding principles for participatory action research with youth. New Dir Youth Dev. 2009;2009(123):19-34, 11. Epub 2009/10/16. doi: 10.1002/yd.312. PubMed PMID: 19830799.
102. Kearney S, Leung L, Joyce A, Ollis D, Green C. Applying systems theory to the evaluation of a whole school approach to violence prevention. Health Promot J Austr. 2016;27(3):230-5. Epub 2016/10/11. doi: 10.1071/he16046. PubMed PMID: 27719735.
103. Atkinson J, Wells R, Page A, Dominello A, Haines M, Wilson A. Applications of system dynamics modelling to support health policy. Public Health Research & Practice.
104. Harvey J. Evaluation Cookbook. Edinburgh, UK Heriot-Watt University (Institute for Computer Based Learning), 1998.
105. Hargreaves MB. Evaluating System Change: A Planning Guide. Princeton, NJ: 2010.

106. Sterling EJ, Zellner M, Jenni KE, Leong K, Glynn PD, BenDor TK, et al. Try, try again: Lessons learned from success and failure in participatory modeling. Elementa: Science of the Anthropocene. 2019;7. doi: 10.1525/elementa.347.

Figure 1

Click here to access/download;Figure;Lee GY_PM evaluation framework scoping review_FIGURE 1.tif

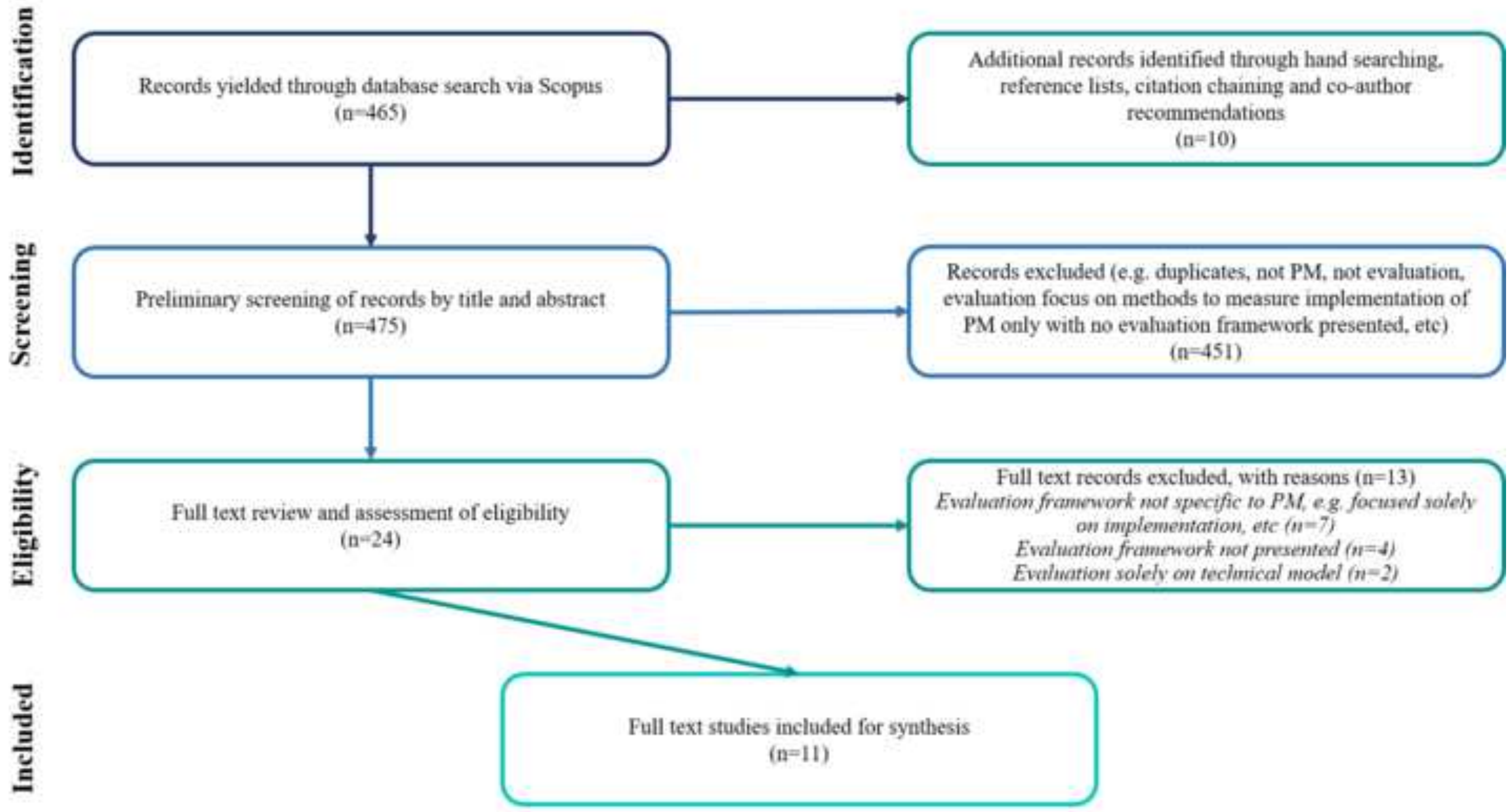

Figure 2

Click here to access/download;Figure;Lee GY_PM evaluation framework scoping review_FIGURE 2.tif

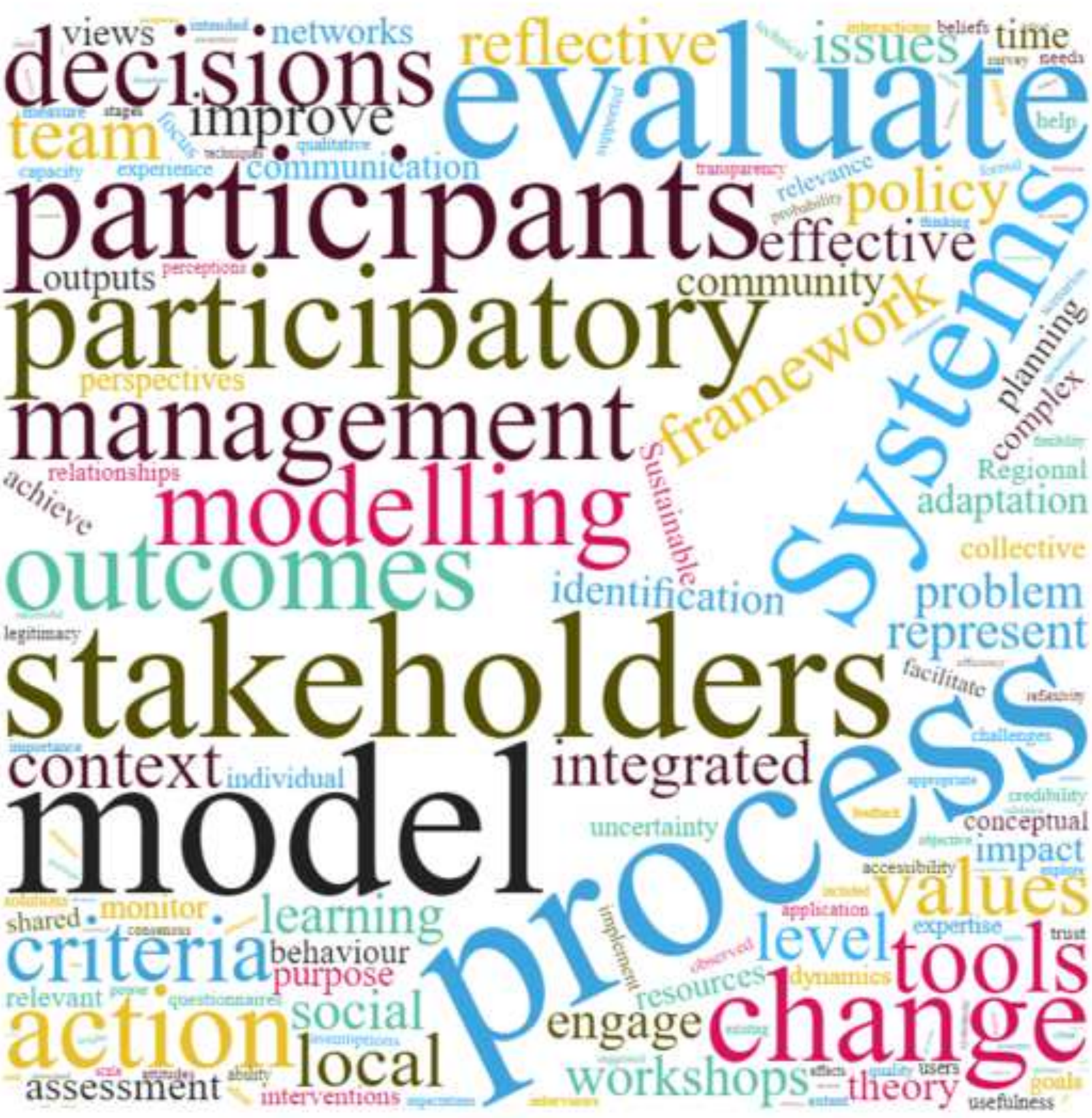

Figure 3

Click here to access/download;Figure;Lee GY_PM evaluation framework scoping review_FIGURE 3.tif

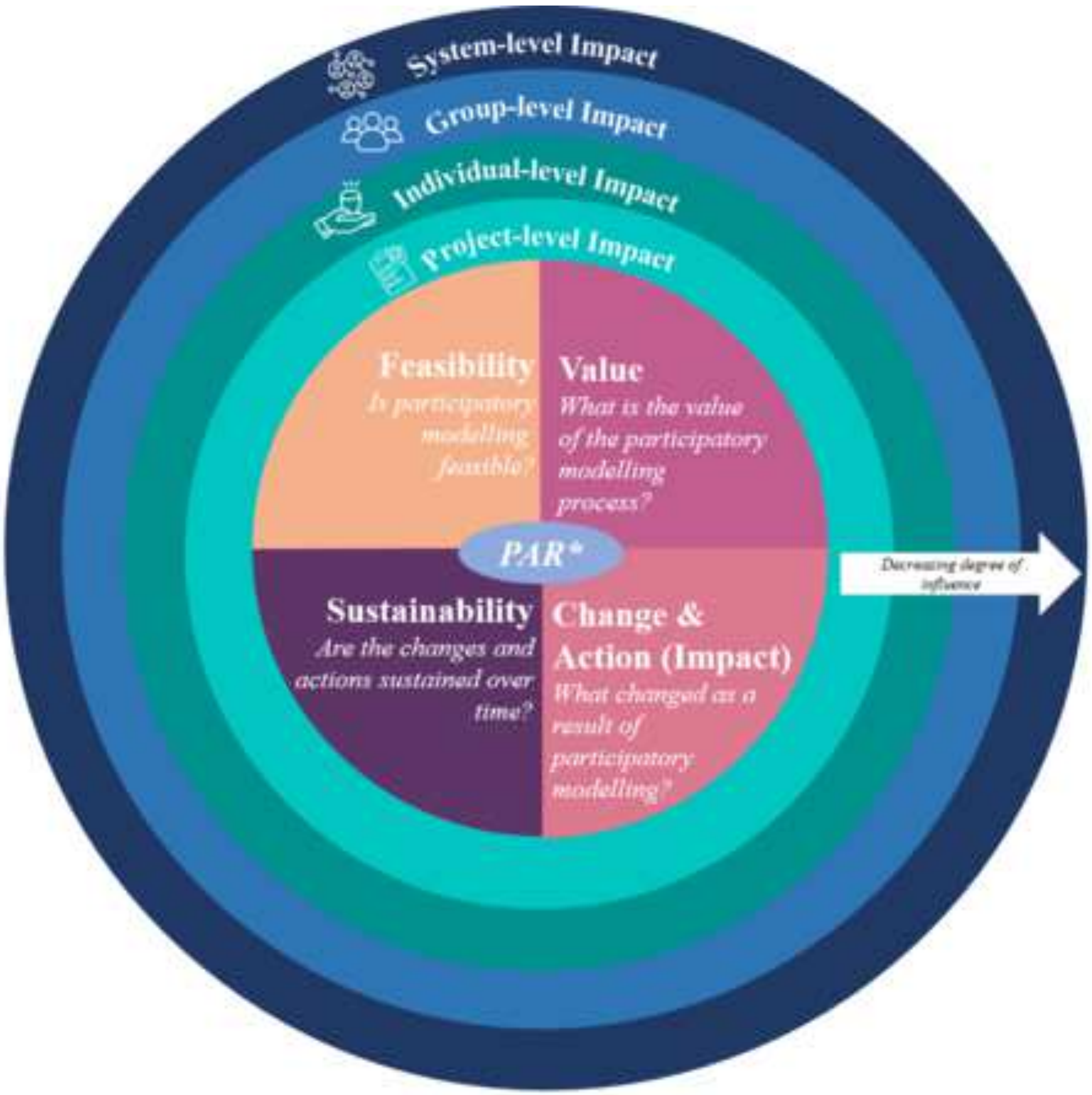

*PARTICIPATORY ACTION RESEARCH (PAR): *Culture of embedding reflection & action during evaluation processes to improve outcomes and reduce inequities*

Figure 4

Click here to access/download;Figure;Lee GY_PM evaluation framework scoping review_FIGURE 4.tif

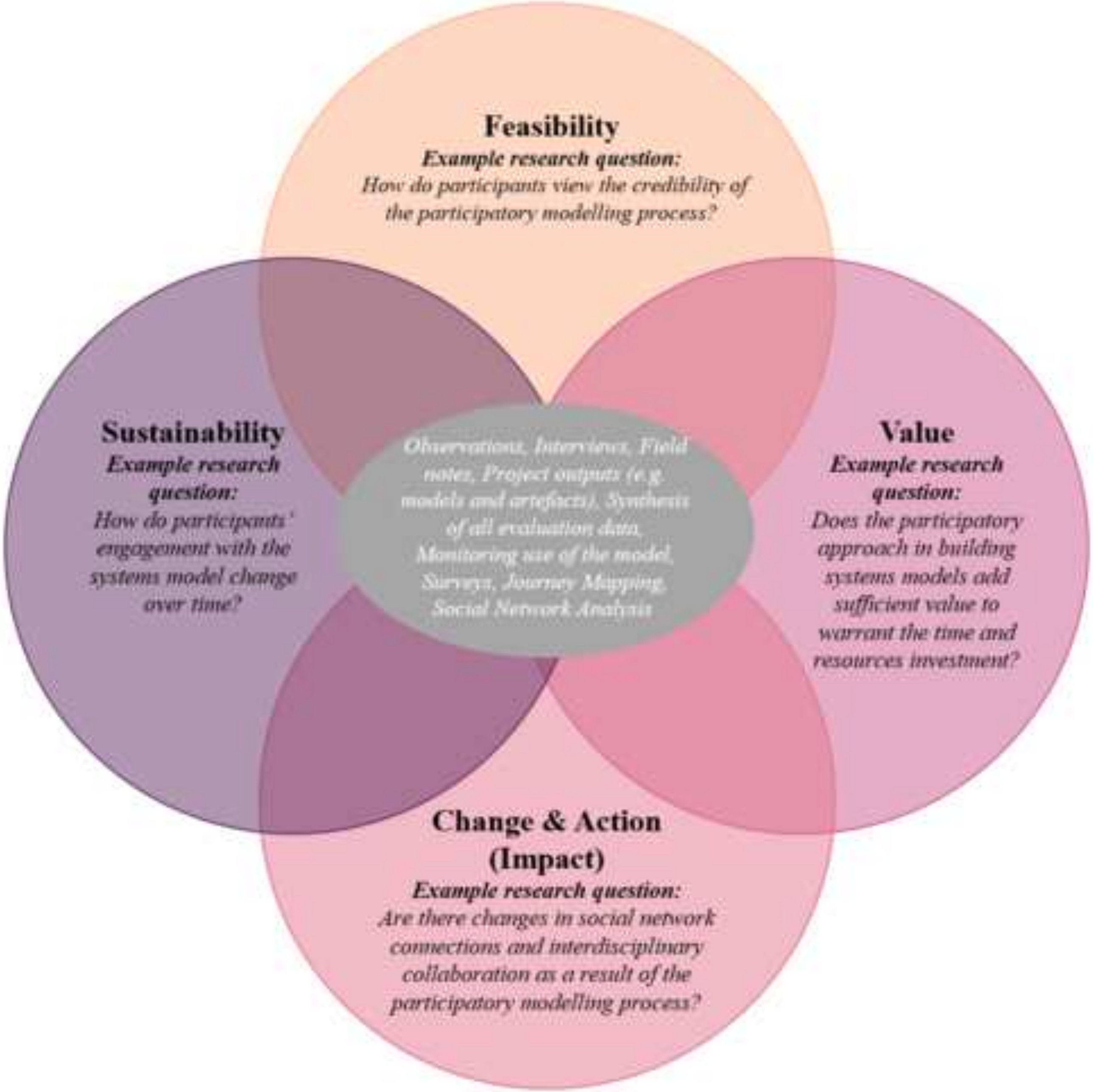

Supplement: Attachment — Submitted filename: PLOS1 rev 2021.pdf [file pone.0266125.s002.pdf]
