# Supplementary material for: Presenting a comprehensive multi-scale evaluation framework for participatory modelling programs: A scoping review
Source: PLoS One. 2022 Apr 22;17(4):e0266125. doi: 10.1371/journal.pone.0266125 (PMC9032404; doi:10.1371/journal.pone.0266125)
Supplement: Attachment — Submitted filename: Response to Reviewers_PONE-D-21-25695R1.docx [file pone.0266125.s005.docx]

Fausto Cavallaro, PhD
Academic Editor
PLOS ONE

23 February 2022

Dear Dr Fausto Cavallaro,

**Re Manuscript – *Presenting a comprehensive multi-scale evaluation framework for participatory modelling programs: A scoping review* (PONE-D-21-25695R1)**

Thank you for your email dated 19 February 2022 providing us with Reviewer’s comments on manuscript (PONE-D-21-25695R1).

Please find attached our revised manuscript, in which we have addressed all comments provided by the Reviewers as outlined in our point-by-point rebuttal below.

**Journal Requirements:**

*1. Please review your reference list to ensure that it is complete and correct. If you have cited papers that have been retracted, please include the rationale for doing so in the manuscript text, or remove these references and replace them with relevant current references. Any changes to the reference list should be mentioned in the rebuttal letter that accompanies your revised manuscript. If you need to cite a retracted article, indicate the article’s retracted status in the References list and also include a citation and full reference for the retraction notice.*

**Our response: Many thanks for your guidance. We have reviewed the reference list to ensure that it is complete and correct. We have made the following changes:**

- **Corrected the author names for References #11, #29, #32. #38, #39, #40, #46, #50, #69, #71, #96.**
- **Corrected the punctuations for References #39, #96.**
- **Updated the registration details of the scoping review for Reference #49.**
- **Added details of the publishing city for Reference #69.**
- **Corrected the title for Reference #77.**
- **Additionally added three new references #100-102.**

**Reviewers’ comments:**

***Reviewer #2:***

*Good job reviewing the paper.*

*My only concern is about the second paper to be published simultaneously with the application example.*

*I am concerned in this case that there is no guarantee that both manuscripts will be accepted and published at the same time. Many journals would discourage this kind of two-part submission, but I would leave this to the PLOS editors to decide. It would be certainly nice if the paper would be self-sufficient.*

*A few minor notes:*

1. *On the title page for three co-authors it is said that: “These authors contributed equally to this work.” Sounds a bit strange. And how did the other authors contribute?*
2. *First sentence of the Abstract says: "Systems modelling and simulation can... better manage system challenges". Not sure what did you mean to say. How do you manage system challenges with modeling?*

**Our response: Thank you for your kind feedback. We agree that the two papers would be best presented together, and thus have requested PLOS ONE to publish simultaneously if this will not substantially delay the publication of this manuscript. Regardless of the journal’s decision, we will ensure that the two papers appropriately reference each other. We have left a note for the PLOS ONE Academic Editor for further consideration.**

**To address your additional notes:**

1. **We felt that the tremendous contributions of Ian Bernard Hickie, Jo-An Occhipinti, and Yun Ju Christine Song in supporting the writing and reviewing of the presented manuscript should be jointly recognized, providing merit in co-second authorship.**
2. **Thank you for your feedback. We have modified this sentence to say “…to support decision making, better managing systems challenges.”**

***Reviewer #3:***

*Thank you for carefully addressing my comments. I think the paper has greatly improved. I just have a few minor issues.*

1. *The evaluation framework that you have developed includes a time dimension; some of the criteria refer to ex ante assessments (e.g. criterion 2), some to ex durante (e.g.criterion 3) and others ex post (e.g. e.g. criterion 25). I think it would improve the evaluation framework if this time dimension is included explicitly, so basically showing what questions need to be asked at what moment in time?*
2. *Not all criteria are clear. E.g. criterion 11: What are facilitators and barriers (e.g. inemtives)? facilitators and barriers for what?*
3. *p4-193 this sentence is unclear :" Stakeholders who have a ‘stake’ in the system, including those who are a part of the local context are viewed as important communication agents to decision makers, facilitating a positive movement whereby stakeholder involvement is perceived as almost necessary – shifting scientists away from working independently to develop systems models [21]."*
4. *p8-180: systematic reviews also use a protocol...?*
5. *p16-339: this sentence is unclear " There were two papers that did not explicitly consider the multi-value perspectives integrated within PM [54, 56].*
6. *p36-514: "In other words, knowledge that is acquired from evaluation outcomes can be applied prospectively throughout the PM process, rather than retrospectively reflecting on before-after measurements." Can you say a bit more about how your framework can be used pro-actively, for the design of PM processes? That would be useful!*

**Our response: Thank you for your thorough review.**

1. **Though we agree that the evaluation framework includes some reference to a time dimension, after careful consideration, we prefer to keep our evaluation framework as it is currently presented. We do explicitly mention the events around which the evaluation criteria can potentially be applied (p29-406), however, it is our intention that the evaluation framework be flexible and adaptive to participatory modelling programs of different structure and duration (as referenced in p7-173, p28-375, p34-475, p36-526, p38-561).**
2. **Thank you for your feedback. We have carefully reviewed all evaluation criteria to ensure clarity and comprehension.**
3. **We have rephrased the sentence (p4-193) to now read [*changes denoted in orange*], “By recognizing the interdependencies within complex systems, diverse stakeholder groups including those who are part of the system being modelled are viewed as important change agents. The involvement of stakeholders is necessary not only for their knowledge contributions but also for their key role in coordinating the implementation of strategic system improvements – hence the value in shifting scientists away from working in isolation to develop systems models [21]."**
4. **To clarify, we do not say that systematic reviews do not use a protocol (p8-180). Rather, we have explicitly stated that traditional literature searches are not routinely informed by an a priori protocol. We have modified our sentence in our manuscript to make this distinction clearer to now read [*changes denoted in orange*], “Scoping reviews also differ from non-systematic literature searches as they are routinely informed by an a priori protocol, …”**
5. **We have rephrased the sentence (p16-339) to now** **read [*changes denoted in orange*], “There were two papers that did not explicitly consider the multi-value perspectives integrated within PM, such as the consideration of the multiple levels of examination (e.g., diverse stakeholder perspectives, project *vs* system level, short *vs* long-term observation, etc) [54,56].”**
6. **We have added an additional sentence (p36-514) to now read** **[*changes denoted in orange*]: "In other words, knowledge that is acquired from evaluation outcomes can be applied prospectively throughout the PM process, rather than retrospectively reflecting on before-after measurements. Through principles of PAR, the proposed evaluation framework embeds continuous cycles of reflection to facilitate shared learning and iterative refinement of processes throughout PM implementation."**
